# Expression, oncological and immunological characterizations of BZW1/2 in pancreatic adenocarcinoma

**DOI:** 10.3389/fgene.2022.1002673

**Published:** 2022-10-04

**Authors:** Jiachen Ge, Senmao Mu, Erwei Xiao, Guangjin Tian, Lianyuan Tao, Deyu Li

**Affiliations:** Department of Hepatobiliary Surgery, Henan Provincial People’s Hospital, People’s Hospital of Zhengzhou University, Zhengzhou, China

**Keywords:** pancreatic adenocarcinoma, BZW1, BZW2, tumor microenvironment, prognosis, tumor infiltrating immune cell

## Abstract

**Background:** Despite the progress in early diagnosis and treatment, prognosis of pancreatic adenocarcinoma (PAAD) is still poor. Basic leucine zipper and W2 domain-containing protein 1 (BZW1) and protein 2 (BZW2) are attached to the basic leucine zipper (bZIP) superfamily. Recently, BZW1 was identified as an important role in glycolysis of PAAD. However, the comprehensive reports about BZW1/2 in PAAD are not sufficient.

**Methods:** RNA-seq data in the Cancer Genome Atlas (TCGA) and Gene Expression Omnibus (GEO) databases were retrospectively analyzed. We explored the expression of BZW1/2 in PAAD tissues and the associations between BZW1/2 and prognosis. In addition, the potential roles of BZW1/2 in tumor microenvironment (TME) of PAAD were analyzed. Finally, clinicopathological data of 49 patients with PAAD in our institution were collected. Immunohistochemistry was used to determine the expression of BZW1/2 in PAAD samples.

**Results:** BZW1 and BZW2 were upregulated in PAAD tissues compared to normal tissues (*p* < 0.05). The expression of BZW1/2 were not significantly correlated with gender, grade and stage of PAAD (*p* > 0.05). High expression of BZW2 was an independent predictor for poor prognosis of PAAD (HR 1.834, 95%CI 1.303–2.581, *p* = 0.001). And a nomogram to predict overall survival (OS) of PAAD was established with a C-index of 0.685. BZW1 and BZW2 expression were positively associated with T cell mediated immune response to tumor cell and Th2 cells in xCell database. Tumor Immune Single-Cell Hub (TISCH) analyses indicated that BZW1 and BZW2 were mainly expressed in B cells and malignant cells. External cohort furtherly validated that high expression of BZW1 and BZW2 were predictors for poor prognosis of PAAD.

**Conclusion:** We found that BZW1 and BZW2 are highly expressed in malignant cells and B cells in the TME of PAAD. BZW2 is an independent predictor for OS of PAAD. BZW1 and BZW2 expression are positively associated with T cell mediated immune response to tumor cell and Th2 cells in PAAD.

## Introduction

Current evidence suggests that the incidence of pancreatic adenocarcinoma (PAAD) has increased apparently in recent years. In the USA, PAAD has become the 10th newest cancer in men and the eighth in women (Siegel et al., 2022). Despite the progress in early diagnosis and standard treatment, the prognosis of PAAD remains poor, with a 5-year overall survival rate of about 10% (Mizrahi et al., 2020). This may be due to resistance to standard therapies, including surgery, chemotherapy and radiotherapy. With the development of precise and targeted therapy, the emerging immunotherapies might successfully improve the prognosis of PAAD (Yin et al., 2022). Given the intricacy of PAAD tumor microenvironment (TME) characterized by fibrosis and poor vascularization, the efficiency of anti-tumor therapy is probably disturbed (Truong and Pauklin, 2021).

Basic leucine zipper and W2 domain-containing protein 1 (BZW1, BZAP45) and protein 2 (BZW2, 5MP1) are important members of the basic leucine zipper (bZIP) superfamily (Mitra et al., 2001; Singh et al., 2011). BZW1 and BZW2 genes encode a 45 kDa protein containing an N-terminal bZIP domain and a C-terminal W2 domain, and are highly expressed in bronchial epithelial cells and placenta, respectively (Hiraishi et al., 2014). As a transcription factor, BZW1 is identified as a conserved regulator during the G1/S transition (Mitra et al., 2001). Moreover, BZW2 is associated with tumorigenesis maintenance and cell-cell adhesion *via* translation initiation and cadherin binding, respectively (Guo et al., 2014). Currently, the roles of BZW1/2 in PAAD have been largely understudied. As a paralog of BZW1, we hypothesized that BZW2 might play a similar role in the progression of PAAD. In this study, we tried to analyze the expression profiles of BZW1/2 and their immunological characteristics in PAAD samples *via* public database. Furthermore, PAAD tissue samples were collected and protein expression of BZW1/2 and prognosis of PAAD were analyzed in an external validation cohort.

## Methods

### Sample collection and pan-cancer analysis

Clinical, pathological and gene-expression data were acquired from the Cancer Genome Atlas (TCGA, https://portal.gdc.cancer.gov/) and Gene Expression Omnibus (GEO, https://www.ncbi.nlm.nih.gov/) databases. Patients who were diagnosed as PAAD with pathological evidence were included in this study. Samples from patients with pancreatic neuroendocrine or metastatic neoplasms were excluded from this study. Normalized pan-cancer datasets, TCGA TARGET GTEx (PANCAN, N = 19131, G = 60499), were downloaded from the UCSC database (https://xenabrowser.net/). Samples derived from solid tissue normal, primary solid tumor, normal tissue, primary blood derived cancer-bone marrow and primary blood derived cancer-peripheral blood were selected, and gene-expression data of BZW1 (ENSG00000082153) and BZW2 (ENSG00000136261) in each sample were extracted. Carcinoma sample number less than 3 was eliminated. As a result, 34 species of carcinoma were incorporated into the pan-cancer analysis.

### Protein levels of BZW1/2 in PAAD samples

Immunohistochemical staining images of PAAD and normal tissues were collected from the Human Protein Atlas (HPA, https://www.proteinatlas.org/) (Colwill and Gräslund, 2011). The HPA could provide normal and pathological human tissue images stained by immunohistochemical sections. Protein levels of BZW1 and BZW2 in normal and PAAD tissues were explored in the HPA. Next, the differential expression of BZW1 and BZW2 proteins between normal and PAAD tissues was analyzed by clinical proteomic tumor or analysis consortium (CPTAC, http://ualcan.path.uab.edu/analysis-prot.html) database (Chandrashekar et al., 2017).

### Roles of BZW1/2 in PAAD characteristics and prognosis

The mRNA expression data of BZW1 and BZW2 were downloaded from TCGA database. The mRNA profiles were standardized by log_2_ (x+0.001) transformation for further analysis. Follow-up was conducted from the data of tumor diagnosis, and the end was data of all-cause death. The recorded age, gender, grade, American Joint Committee on cancer (AJCC) stage, TNM stage, overall survival (OS) time and status of the samples were collected. Patients were grouped based on gender, grade, AJCC stage and TNM stage. The differences in BZW1 and BZW2 expression among groups were analyzed. Survival differences between the groups were also analyzed. Clinicopathological variables that were significantly correlated with prognosis were used to establish a prognostic model. An external cohort, GSE85916, was used to validate the effects of BZW1 and BZW2 on the prognosis of PAAD patients.

### Similar gene detection analysis

Similar gene detection module on the Gene Expression Profiling Interactive Analysis (GEPIA) website (http://gepia.cancer-pku.cn/detail.php) was used to explore genes with similar expression patterns to BZW1 and BZW2 of PAAD in TCGA dataset (Tang et al., 2017). The top 100 correlated genes of BZW1 and BZW2 were listed in descending order according to the Pearson correlation coefficient (PCC) values, which reflected the correlation intensity between variables (0.0–0.2, extremely poor; 0.2–0.4, poor; 0.4–0.6, moderate; 0.6–0.8, strong; >0.8, extremely strong).

### Functional enrichment analysis

To better understand the potential functions of BZW1 and BZW2, Gene ontology (GO) annotations and Kyoto Encyclopedia of Genes and Genomes (KEGG) pathway annotations were performed by the Database for Annotation, Visualization, and Integrated Discovery (DAVID 6.8, https://david.ncifcrf.gov/). Cellular component (CC), molecular function (MF) and biological process (BP) categories were explored in GO analyses. Statistical significance was set as *p* < 0.05. The *p* values of the top five pathways were sorted in ascending order and displayed.

### Gene set variation analysis (GSVA)

Gene lists of pancreatic cells and immune processes were acquired from Molecular Signatures Database (MSigDB v7.5.1, http://www.gsea-msigdb.org/gsea/msigdb/). We calculated the functional enrichment score of each PAAD sample. PCCs were used to evaluate the correlation between BZW1/2 expression and pancreatic cells or immune processes.

### Establishment of the protein-protein interaction (PPI) network

It is well-established that the STRING database could be used to collect, score and integrate publicly available sources of PPI information (von Mering et al., 2005). The top 100 correlated genes were selected and incorporated to establish PPI networks. The network was constructed with a medium confidence at 0.400.

### Tumor immune single-cell hub analysis

Tumor Immune Single-Cell Hub (TISCH, http://tisch.comp-genomics.org/home/) could provide detailed cell-type annotation at the single-cell level, and enabled the exploration of TME about different cancer types. The PAAD_CRA001160 dataset was selected to compare BZW1 and BZW2 expression in three main cell types, including immune cells, stromal cells and malignant cells. PAAD_CRA001160 dataset in TISCH contained single-cell RNA-seq of 57443 individual pancreatic cells from 35 primary PAAD patients (Peng et al., 2019).

### ESTIMATE-stromal-immune score analysis

We further analyzed the roles of BZW1 and BZW2 in the TME. The Estimation of Stromal and Immune Cells in Malignant Tumors Tissues using Expression Data (ESTIMATE) algorithm was used to detect the fractions of infiltrating stromal and immune cells. And immune and stromal scores could be inferred by ESTIMATE algorithm. Higher immune or stromal components were associated with higher immune or stromal scores. The associations of BZW1/2 genes and ESTIMATE-Stromal-Immune score were explored.

### Tumor-infiltrating immune cell abundance analysis

The correlations of BZW1/2 genes and tumor-infiltrating immune cell (TIIC) in PAAD samples were analyzed. The PCCs were calculated by CIBERSORT (https://cibersort.stanford.edu/), xCell (https://github.com/dviraran/xCell), MCP counter (https://github.com/ebecht/MCPcounter), Estimate the Proportion of Immune and Cancer cells (EPIC, https://gfellerlab.shinyapps.io/EPIC_1-1/), Tumor Immune Estimation Resource (TIMER, https://cistrome.shinyapps.io/timer/), quanTIseq (http://icbi.i-med.ac.at/software/quantiseq/doc/index.html) and immunophenoscore (IPS, https://tcia.at/home). The computational methods mentioned were applied to estimate TIIC abundance in PAAD samples. The associations between BZW1/2 expression and TIIC abundance were analyzed.

### Cell lines and culture

The human pancreatic ductal epithelium cell line HPDE6-C7 and two human pancreatic cancer cell lines, including MiaPaCa-2 and Panc-1, were obtained from the American Type Culture Collection (ATCC, Manassas, VA, USA). The cell culture medium was Dulbecco’s Modified Eagle Medium (DMEM) (Invitrogen, Carlsbad, CA, USA) supplemented with 10% fetal bovine serum (FBS) (Invitrogen). Cells (10^5^) were grown in a humidified 5% CO_2_ incubator at 37°C.

### Real-time quantitative polymerase chain reaction (RT-qPCR)

Total RNA was extracted from cell lines using TRIzol reagent (Invitrogen, USA)and reverse transcribed into cDNA *via* the PrimeScript RT Reagent Kit with gDNA Eraser (Takara, Japan). RT-qPCR was carried out *via* TB Green Premix Ex Taq II (Takara). In this respect, the RNA template and its primer were incubated at 70°C for 10min, and cooled on ice for 5min. The complex mixture was incubated at 42°C for 50min, and then heated at 70°C for 15min. The mixed reaction mixtures were amplified for 40 cycles with the following procedure, 95°C for 10s, 60°C for 10s and 72°C for 20s. Expression of mRNA was calculated by 2^−ΔΔCt^ method. GAPDH was used to normalize the results of RT-qPCR. The primer sequences were as following: GAPDH, GGAGCGAGATCCCTCCAAAAT(F), GGCTGTTGTCATACTTCTCATGG(R); BZW1, AAGAGAGGTTTGACCCTACTCAG(F), CTGCATATCGACGGTAATCAAGT(R); BZW2, CTAACAGGCCAGCGGTTCAAA(F), GGACAAGTGTATCCCTGAAGACT(R).

### Immunohistochemistry of BZW1 and BZW2 in PAAD samples

To further clarify the expression of BZW1 and BZW2 in PAAD samples, we retrospectively collected the clinicopathological data of 49 patients who underwent needle biopsy or radical surgery in the Department of Hepatobiliary Surgery at Henan Provincial People’s Hospital from June 2018 to May 2021. Patients were asked to receive systematic chemotherapy post-operation if circumstances permitted. After discharge, they were requested to periodic follow-up. Immunohistochemistry staining was performed to analyze the expression of BZW1 and BZW2 in tumor specimens. BZW1-(#ab85090) and BZW2-(#ab254772) antibodies were purchased from Abcam company. The EnVision two-step method was used to stain the specimen. The expression of BZW1 and BZW2 were recorded by light microscopy and evaluated by two pathologists independently. The correspondence rules of staining intensity and scoring standard were as following: uncolored, 0; light yellow, 1; yellow, 2; brown, 3. Positive expression region percentage criteria were as following: 0–30%, 0; 30–60%, 1; >60%, 2. After calculating the total points, samples were grouped into low-expression (0–2 points) and high-expression (three to five points) groups. The Pearson correlation coefficient between the two pathologists was 0.87.

### Statistical analysis

All statistical analyses in this study were conducted by R software (version 4.1.0). Comparison analyses among expression quantities of BZW1 and BZW2 were performed by student’s t test, Mann-Whitney *U* test or variance analysis. The Kaplan-Meier method was used to analyze survival difference between low- and high-expression level groups. Significance of the correlation between the two groups was tested by Pearson correlation analysis. OS was calculated by the Kaplan-Meier method, and the survival differences were compared by the Log-Rank test. Univariate and multivariate cox proportional hazards regression methods (backward selection) were used to identify clinicopathologic variables significantly associated with OS. The optimal cut-off values between low- and high-expression groups were determined by X-tile software (version 3.6.1). The PPI network was visible *via* Cytoscape software (version 3.9.1). R package “regplot” was used to construct the nomogram. A two-tailed *p* value less than 0.05 was statistically significant unless otherwise mentioned.

## Results

### BZW1/2 were upregulated in PAAD

**FIGURE 1:**
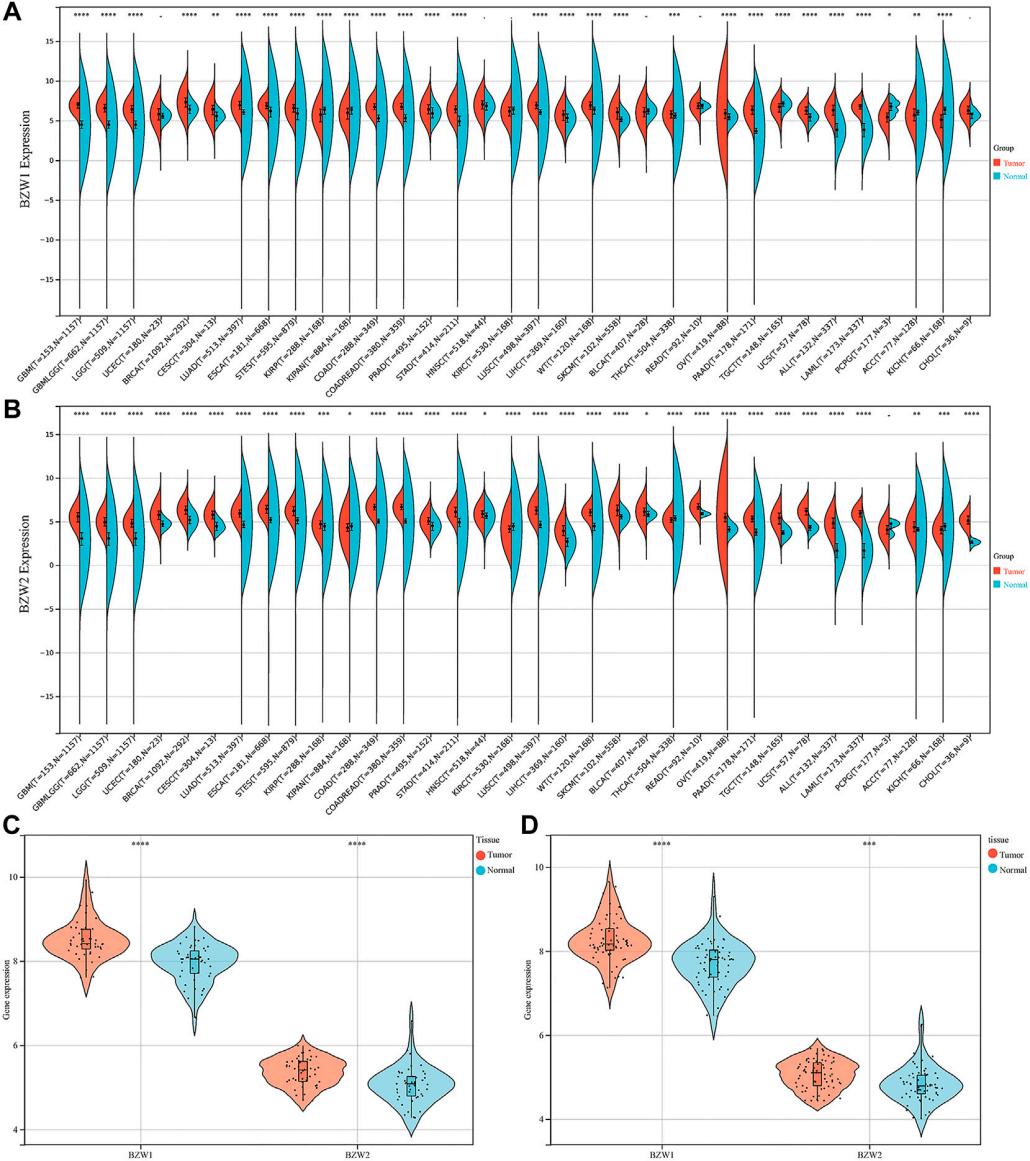
BZW1 **(A)** and BZW2 **(B)** expression in pan-cancer. Expression of BZW1 and BZW2 was upregulated in tumor tissues in GSE28735 **(C)** and GSE62452 **(D)** cohorts. *p < 0.05, **p < 0.01, ***p < 0.001, ****p < 0.0001.

**FIGURE 2:**
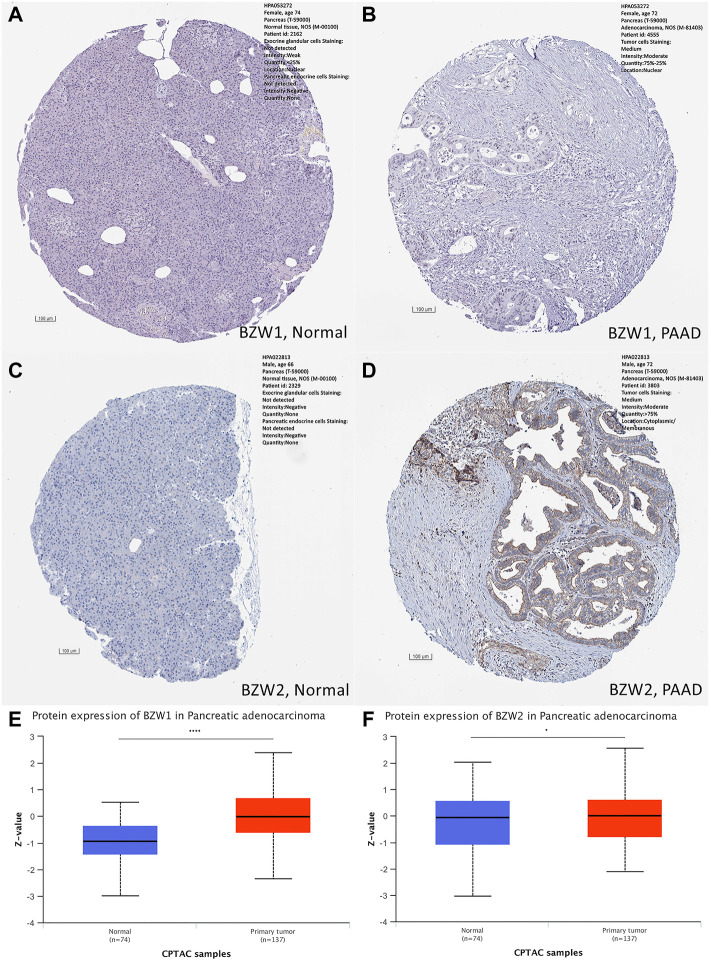
Immunohistochemistry staining in normal and PAAD tissues, respectively. Compared to normal tissues, the expression levels of BZW1 and BZW2 were upregulated in PAAD tissues **(A,B,C,D)**. Compared to normal tissues, expression levels of BZW1 **(E)** and BZW2 **(F)** proteins were significantly higher in PAAD tissues based on CATAC database.

Expression data about 34 types of carcinomas was acquired from the UCSC database. Mann-Whitney *U* test was used to make differential expression analyses. A significant expression difference of BZW1 between normal and tumor samples was observed in 28 species. Among them, up-regulated expression of BZW1 in tumor tissue was detected among 22 types of carcinomas, including GBM, GBMLGG, LGG, BRCA, CESC, LUAD, ESCA, STES, COAD, COADREAD, PRAD, STAD, LUSC, LIHC, WT, SKCM, THCA, OV, PAAD, UCS, ALL and LAML (*P* all <0.001). And down-regulated expression of BZW2 was detected among 6 types of carcinomas, including KIRP, KIPAN, TGCT, PCPG, ACC and KICH (*Ps* < 0.05, Figure 1A, Supplementary Table S1). Highly expressed BZW2 was observed in 30 species of carcinomas, including GBM, GBMLGG, LGG, UCEC, BRCA, CESC, LUAD, ESCA, STES, KIRP, KIPAN, COAD, COADREAD, PRAD, STAD, HNSC, LUSC, LIHC, WT, SKCM, BLCA, READ, OV, PAAD, TGCT, UCS, ALL, LAML, ACC and CHOL (*Ps* < 0.05). Moreover, down-regulated expression of BZW2 in tumor tissue was detected among KIRC, THCA and KICH (*Ps*, Figure 1B, Supplementary Table S1). We acquired expression data of two cohorts from the GEO database, GSE28735 and GSE62452. And the results indicated that BZW1 and BZW2 were up-regulated in PAAD compared to normal tissues (*P* all <0.001, Figures 1C,D, Supplementary Table S2). Immunohistochemistry images of BZW1 and BZW2 were obtained from the HPA website. Compared to normal tissues, BZW1 and BZW2 proteins were highly expressed in PAAD tissues using the same antibody (BZW1 antibody: HPA053272; BZW2 antibody: HPA022813) (Figures 2A–D). In the CPTAC database, expression levels of BZW1 and BZW2 in PAAD samples were significantly higher than in normal samples (Figures 2E,F).

### BZW2 was an independent predictor for OS of PAAD patients

**FIGURE 3:**
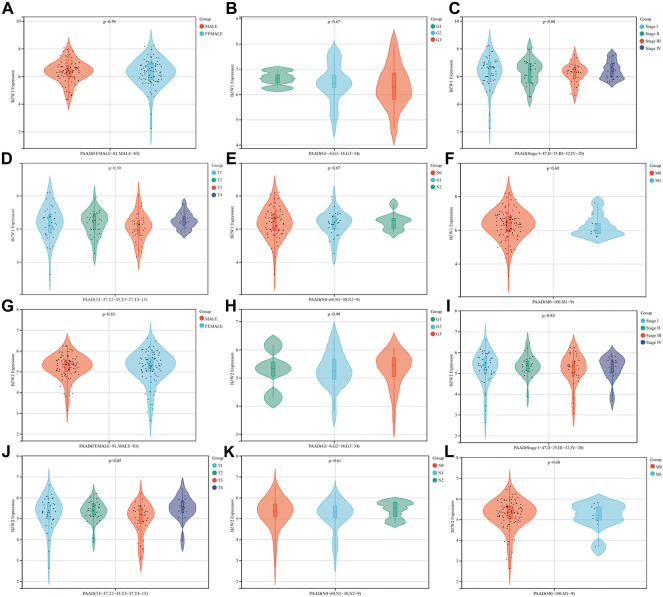
Relationships between BZW1/2 expression and clinicopathological parameters of PAAD. The expression of BZW2 in patients with T3 stage disease was higher than in T2 and T4 stage **(J)**. BZW1 and BZW2 expression were not significantly associated with gender **(A,G)**, grade **(B,H)**, AJCC stage **(C,I)** and TNM stage **(D,E,F,K,L)** of PAAD patients.

The relationships between BZW1/2 expression and clinicopathological parameters were investigated. The results indicated that patients with T3 stage PAAD exhibited higher expression level of BZW2 than T2 and T4 stage (*Ps* < 0.05, Figure 3J, Supplementary Table S3). In addition, BZW1 and BZW2 expression were not significantly associated with gender, grade, AJCC stage and TNM stage of PAAD patients (*Ps* > 0.05, Figure 3A-I, K, L, Supplementary Table S3).

**FIGURE 4:**
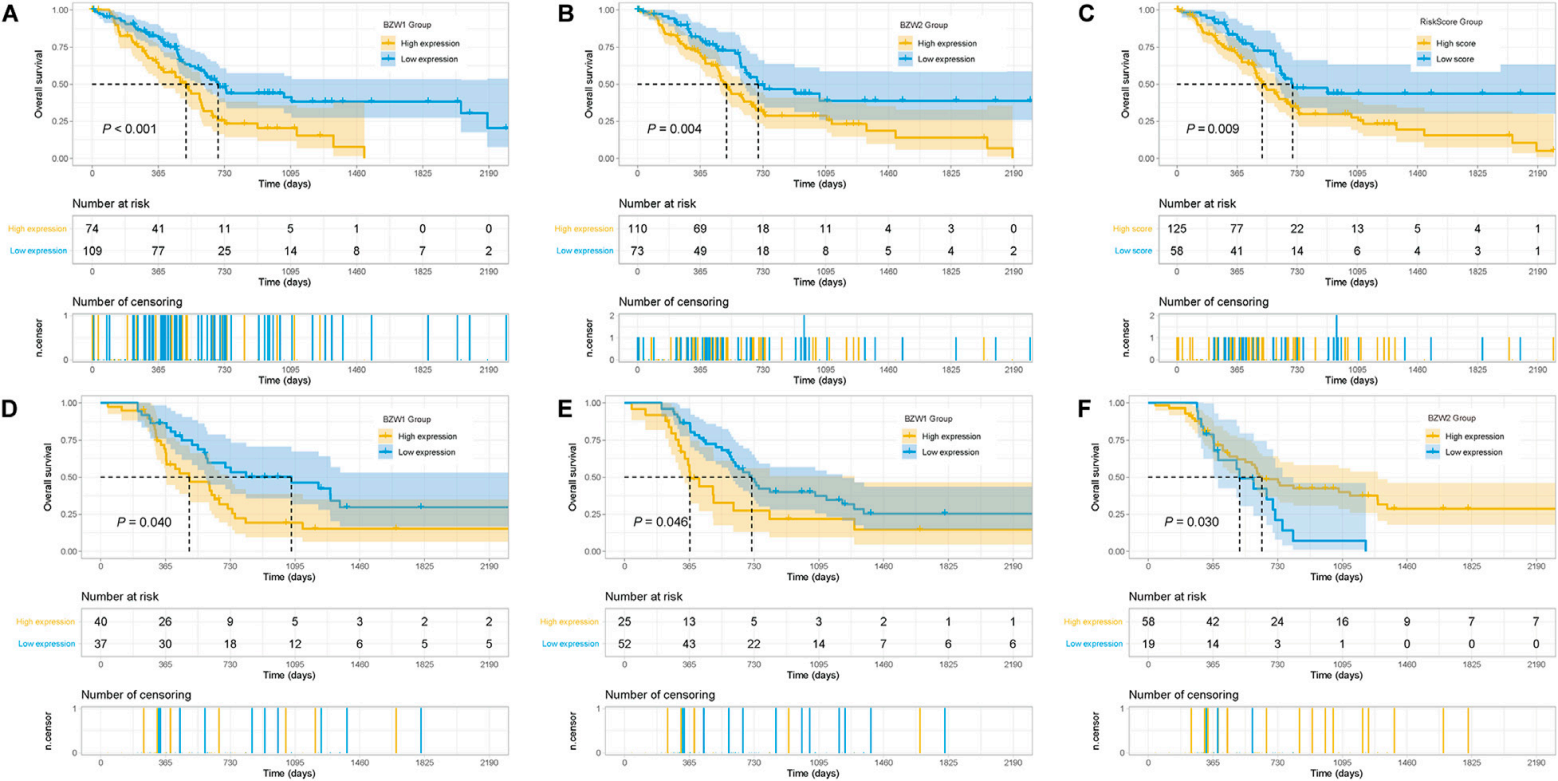
Kaplan-Meier survival curves of different BZW1 and BZW2 expression for TCGA-PAAD patients **(A,B)**. Survival curve of low- and high-RiskScore group for TCGA-PAAD patients **(C)**. Survival curves indicated that patients with high expression of BZW1 (11757867_s_at and 11744775_x_at) and BZW2 (11747677_a_at) suffered poor prognosis in GSE85916 cohort **(D)**: 11757867_s_at, **(E)** 11744775_x_at and **(F)** 11747677_a_at).

**FIGURE 5:**
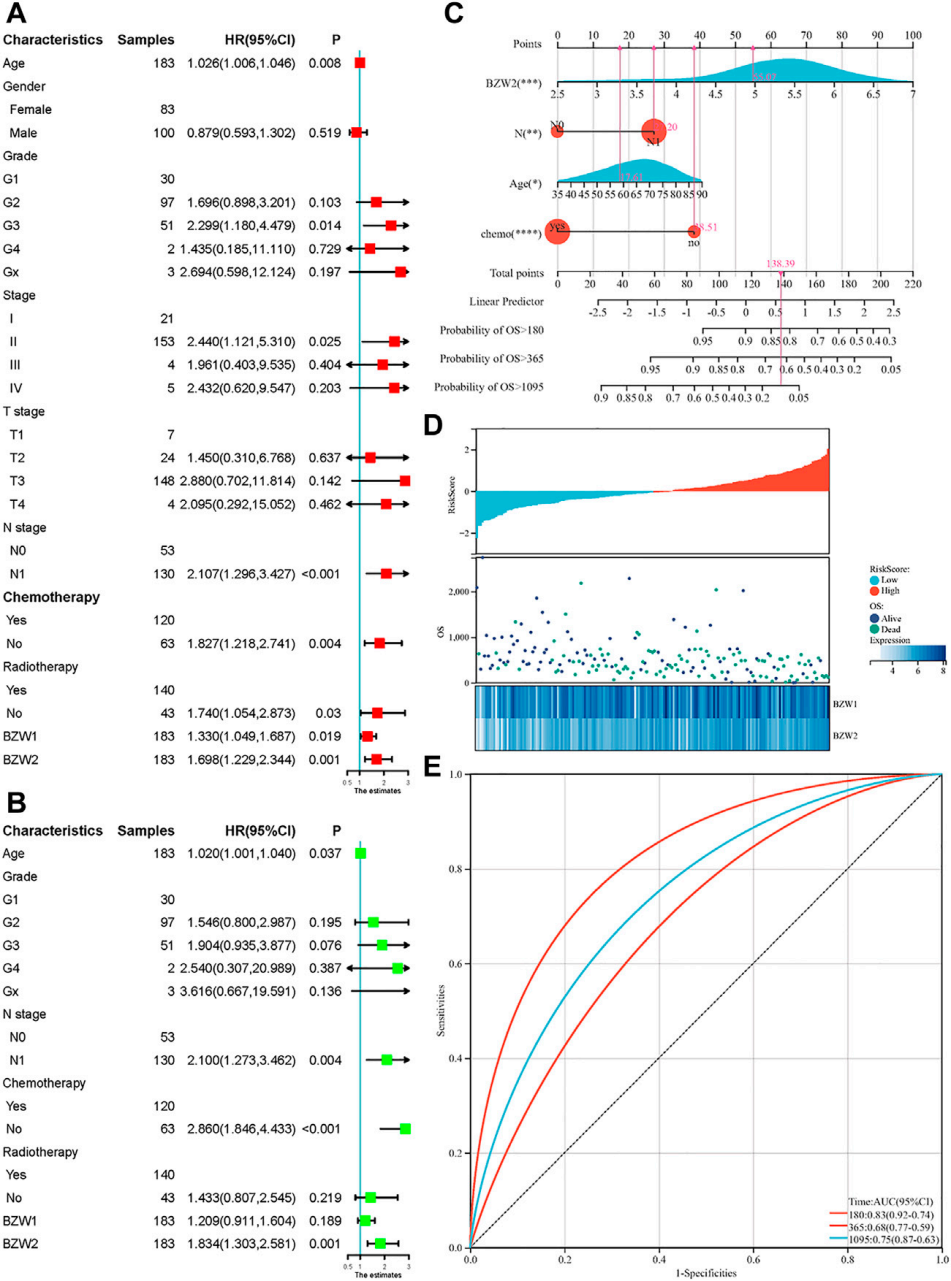
Univariate (A) and multivariate (B) cox regression for OS of PAAD patients. A nomogram was established to predict the OS of PAAD patients based on the independent predictors (C). Heatmap and scatter plot of risk score, survival time and status of all samples (D). ROC curves of the nomogram to predict 180-, 365- and 1095-day survival (E).

Kaplan-Meier survival analysis indicated that PAAD patients with high BZW1 and BZW2 expression had poorer OS than those with low BZW1 and BZW2 levels (*p* < 0.001 for BZW1, *p* = 0.004 for BZW2) (Figures 4A,B). GSE85916 cohort was used to validate the influences of BZW1 and BZW2 expression on prognosis. Survival analyses showed that high expression of BZW1 and BZW2 were significantly associated with short OS for PAAD patients (*p* = 0.040 for BZW1 probe 11757867_s_at, *p* = 0.046 for BZW1 probe 11744775_x_at and *p* = 0.030 for BZW2 probe 11747677_a_at) (Figures 4D–F). Univariate cox regression analysis indicated that age, grade, AJCC stage, N stage, chemotherapy, radiotherapy, BZW1 and BZW2 expression were independent predictors for OS (Figure 5A). Multivariate cox proportional regression showed that older age (HR 1.020, 95%CI 1.001–1.040, *p* = 0.037), N1 stage (HR 2.100, 95%CI 1.273–3.462, *p* = 0.004), no chemotherapy (HR 2.860, 95%CI 1.846–4.433, *p* < 0.001) and higher BZW2 expression (HR 1.834, 95%CI 1.303–2.581, *p* = 0.001) were independently associated with poor OS (Figure 5B). A nomogram for OS of patients with PAAD was established based on these independent predictors. After adding the score corresponding to each factor, the risk score of each patient and the probability of OS longer than 180-, 365- and 1095-day could be calculated (Figure 5C). Survival probability of patients with higher risk score was significantly lower than those with lower risk score (*p* = 0.009, Figure 4C). This nomogram demonstrated a C index of 0.685 (95%CI 0.657–0.713). Heatmap and scatter plot indicated that the survival rate decreased with an increased risk score (Figure 5D). The receiver operator characteristic curves (ROCs) of 180-,365- and 1095-day survival were plotted in Figure 5E.

### Similar gene analyses of BZW1/2

Similar genes module in the GEPIA webtool was used to explore similar genes of BZW1 and BZW2 genes. The top similar 100 genes of BZW1 and BZW2 were listed in Supplementary Table S3. The top five highly correlated genes with BZW1 were BZW1P2, NAA50, KPNA4, G2E3 and RAB10 according to the PCC values. And the top five highly correlated genes with BZW2 were CBX3, NIFK, AVL9, PPIAP22 and PPIA. The heatmap in Supplementary Figure S1 indicated the clustering and correlation of similar genes.

### Biological function exploration

**FIGURE 6:**
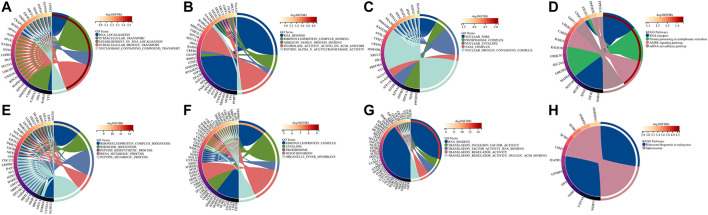
Circos plots displayed the results of GO and KEGG analyses for BZW1, BZW2 and their similar genes. **(A)** BZW1 GO BP. **(B)** BZW1 GO MF. **(C)** BZW1 GO CC. **(D)** BZW1 KEGG. **(E)** BZW2 GO BP. **(F)** BZW2 GO CC. **(G)** BZW2 GO MF. **(H)** BZW2 KEGG.

To further explore the potential biological functions of BZW1 and BZW2, we performed GO and KEGG analyses based on BZW1/2 and their similar genes. The top five enriched BP clusters of BZW1 included RNA localization, intracellular transport, establishment of RNA localization, intracellular protein transport and nucleobase containing compound transport (Figure 6A). The top five enriched MF clusters of BZW1 included RNA binding, ribonucleoprotein complex binding, arrestin family protein binding, hydrolase activity acting on acid anhydride and peptide alpha-N-acetyltransferase activity (Figure 6B). The top five enriched CC clusters of BZW1 included nuclear pore, phosphatase complex, nuclear envelop, NatA complex and nuclear protein containing complex (Figure 6C). Significantly enriched KEGG pathways involved in variations of BZW1 expression included RNA transport, protein processing in the endoplasmic reticulum, AMPK signaling pathway and mRNA surveillance pathway (Figure 6D). The top five enriched BP clusters of BZW2 included ribonucleoprotein complex biogenesis, ribosome biogenesis, peptide biosynthetic process, RRNA metabolic process and peptide metabolic process (Figure 6E). The top five enriched CC clusters of BZW2 included ribonucleoprotein complex, envelope, pre-ribosome, mitochondrion and organelle inner membrane (Figure 6F). The top five enriched MF clusters of BZW2 included RNA binding, translation initiation factor activity, translation factor activity RNA binding, translation regulator activity and translation regulator activity nucleic acid binding (Figure 6G). The enriched KEGG pathways involved in variations of BZW2 expression included ribosome biogenesis in eukaryotes and spliceosome (Figure 6H).

### BZW1/2 expression correlated with pancreatic cells and immune processes

**FIGURE 7:**
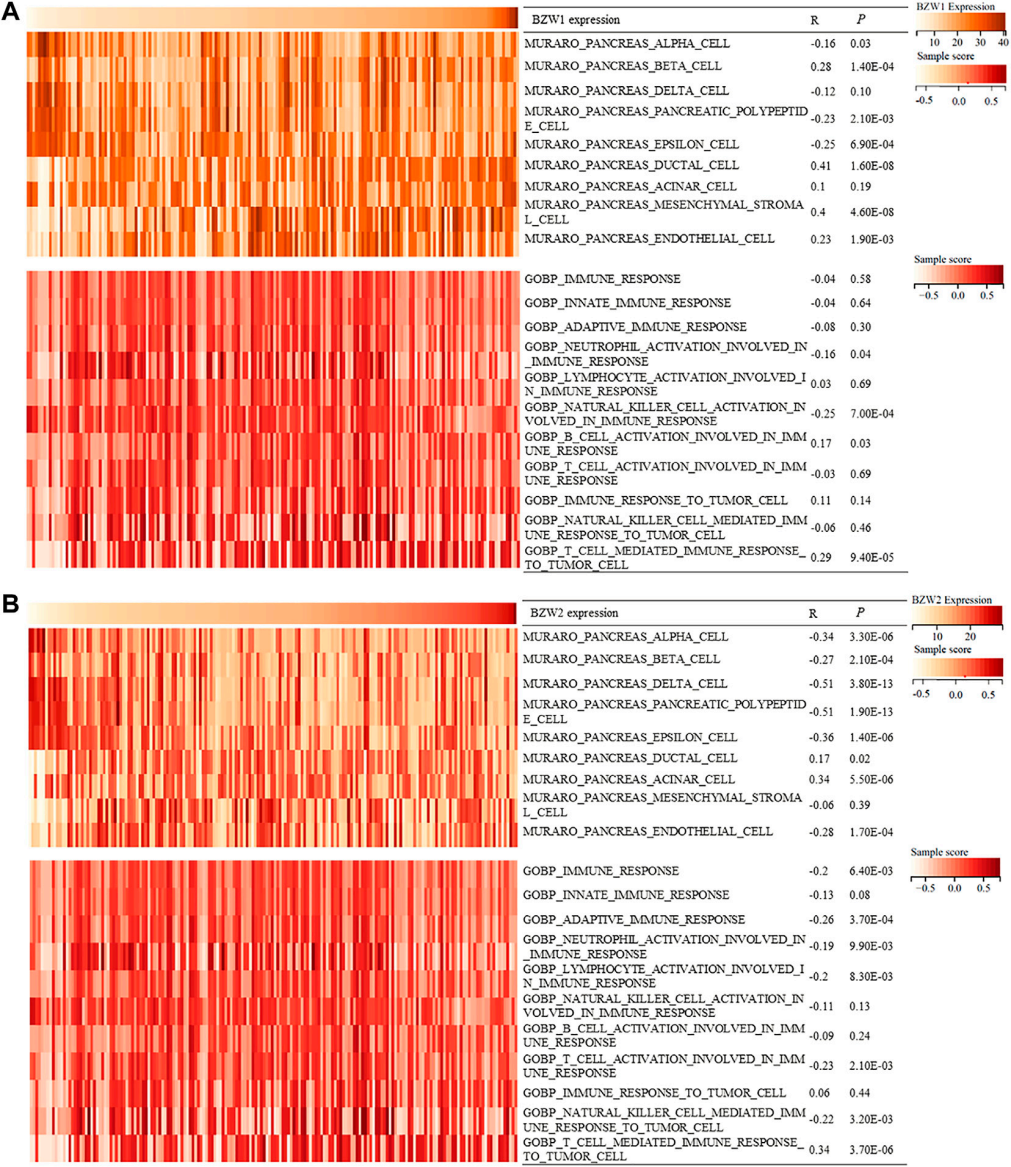
Heatmap showed BZW1/2 expression and enrichment scores of pancreatic cells and immune responses about each sample in each TCGA sample. **(A)** Correlation analyses between BZW1 expression and pancreatic cell or immune response enrichment scores. **(B)** Correlation analyses between BZW2 expression and pancreatic cell or immune response enrichment scores.

During the progression and evolution of the TME, it was highly likely that interactions prevail between PAAD and normal pancreatic cell or immune processes. Hence, we tried to explore the effects of BZW1/2 genes on pancreatic cells and immune processes. GSVA was used to determine the enrichment scores of pancreatic cells and immune processes in TCGA samples. Correlation analysis indicated that BZW1 and BZW2 expression was negatively associated with pancreas alpha cell, pancreatic polypeptide cell and epsilon cell. Moreover, BZW1 and BZW2 expression was positively associated with pancreas ductal cells. In addition, the results of correlation analysis suggested that BZW1 and BZW2 expression was negatively associated with neutrophil activation involved in the immune response but positively correlated with T cell mediated immune response to tumor cells (*Ps* < 0.05, Figures 7A,B).

### Associations between BZW1/2 expression and the TME of PAAD

It was widely acknowledged that the ESTIMATE-Stromal-Immune score could be used to evaluate the composition of immune cells and stromal cells in the TME. In the present study, stromal score, immune score and ESTIMATE score of each sample were calculated. The results indicated that BZW1 expression was positively associated with stromal score and ESTIMATE score. However, BZW2 expression was be not significantly correlated with stromal score, immune score and ESTIMATE score (Table 1 and Supplementary Figure S2).

**TABLE 1:** Correlation of BZW1/2 expression and ESTIMATE-Stromal-Immune score (Pearson).

Gene symbol	Stromal score		Immune score		ESTIMATE score	
	R value	*p* value	R value	*p* value	R value	*p* value
BZW1	0.253	6.57*E*-4	0.078	0.301	0.173	0.021
BZW2	-0.030	0.689	-0.029	0.698	-0.031	0.678

### Correlation between BZW1/2 and immune infiltration in PAAD

We further assessed the correlations between BZW1/2 expression and the types of TIICs. TIICs were evaluated by CIBERSORT, xCELL, MCPcounter, EPIC, TIMER, quanTIseq and IPS methods. These public microarray datasets were used to calculate immune infiltration score of each sample. After filtering the results using *p* > 1.0*E*-5, we found that BZW1 expression was negatively associated with the infiltration of basophils, CD4^+^ Tcm, MEP, NKT and Th1 cells, and positively associated with the presence of CLP, smooth muscle, Th2 cells, neutrophils, T cell CD8, DC, macrophages M1. Moreover, BZW2 expression was negatively correlated with hepatocytes, neurons and Tgd cells, and positively correlated with epithelial cells, keratinocytes, sebocytes, Th2 cells and macrophages M1 (Table 2, Supplementary Figure S3). These results indicated that BZW1 and BZW2 expression influenced TIICs through severalpathways.

**TABLE 2:** Correlations between BZW1/2 expression and TIICs in PAAD (Pearson).

	Negative correlation			Positive correlation		
	Cell type (dataset)	*p* value	R value	Cell type (dataset)	*p* value	R value
BZW1	Basophils (xCELL)	3.394E-9	-0.426	CLP (xCELL)	1.718E-6	0.351
	CD4^+^ Tcm (xCELL)	5.390E-7	-0.366	smooth muscle (xCELL)	3.227E-8	0.401
	MEP (xCELL)	1.155E-13	-0.520	Th2 cells (xCELL)	2.804E-6	0.344
	NKT (xCELL)	1.937E-08	-0.407	neutrophils (MCPcounter)	4.127E-8	0.398
	Th1 cells (xCELL)	7.521E-13	-0.505	neutrophils (quanTIseq)	4.962E-9	0.422
				neutrophils (TIMER)	7.93E-10	0.441
				T cell CD8 (TIMER)	1.450E-16	0.569
				DC (TIMER)	1.655E-15	0.552
				macrophages M1 (quanTIseq)	2.030E-8	0.406
BZW2	hepatocytes (xCELL)	2.062E-10	-0.455	epithelial cells (xCELL)	5.393E-15	0.544
	neurons (xCELL)	2.880E-10	-0.451	keratinocytes (xCELL)	3.884E-10	0.448
	Tgd cells (xCELL)	9.534E-6	-0.326	sebocytes (xCELL)	1.122E-8	0.413
				Th2 cells (xCELL)	2.049E-7	0.379

### PPI network construction and TISCH analysis

**FIGURE 8:**
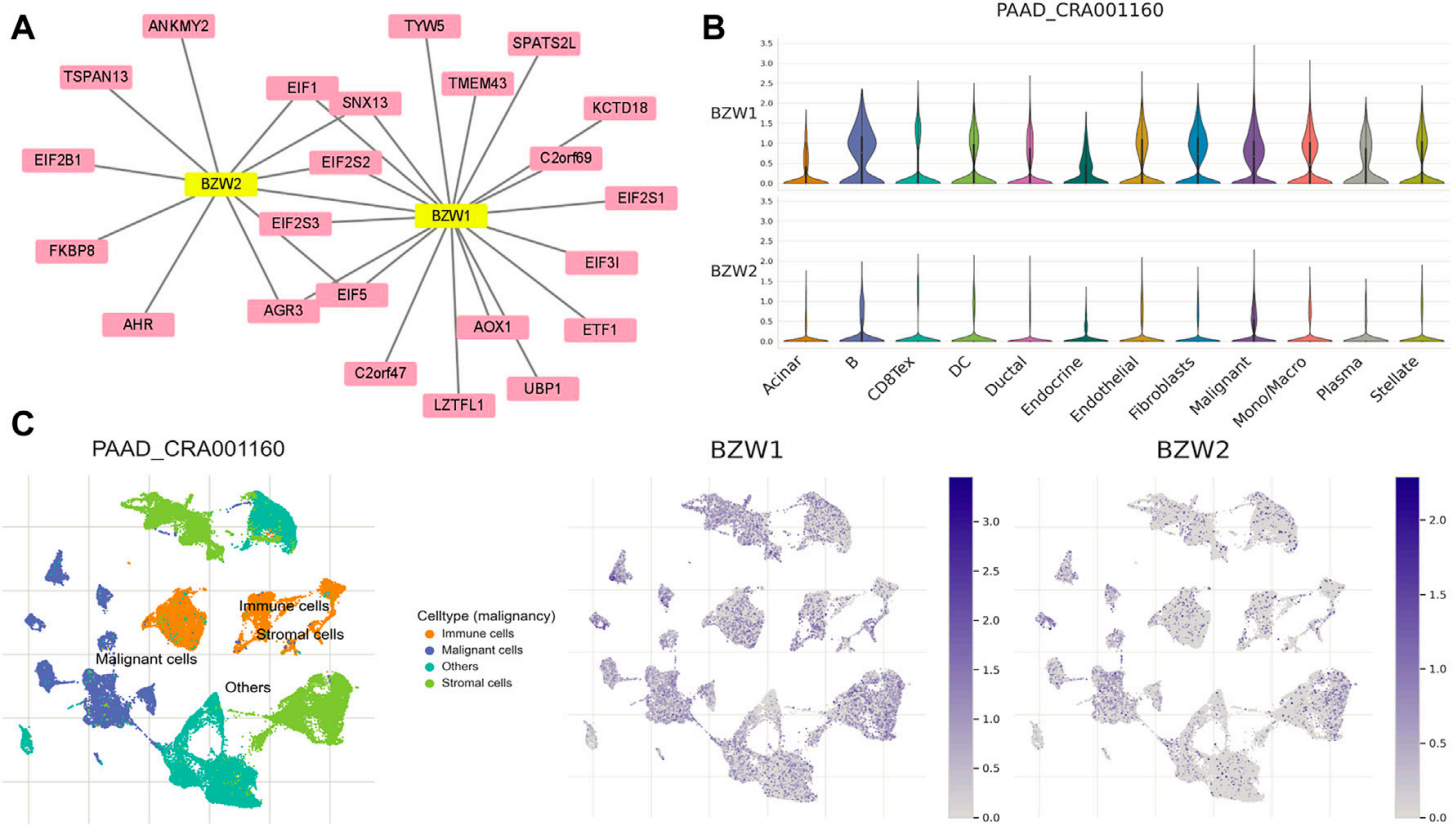
**(A)** PPI network of BZW1- and BZW2-corrected genes based on Cytoscape. **(B)** Violin plots indicated the expression of BZW1 and BZW2 among different cell types in the PAAD_CRA001160 cohort. **(C)** T-distributed stochastic neighbor embedding plots showed the expression of BZW1 and BZW2 in different cell types.

To clarify the co-expression and interactions among of proteins, the PPI network was constructed based on the STRING database (Figure 8A). 18 and 11 proteins were proven to be interacted with BZW1 and BZW2, respectively. The interaction in the PPI network were shown in Supplementary Table S5. AGR3, eIF1, eIF2S2, eIF5 and SNX13 genes were interacted with both BZW1 and BZW2 at the same time. The PAAD_CRA001160 cohort in the TISCH database was used to investigate the relationships between BZW1/2 expression levels and tumor stromal cell infiltrations. Due to the heterogeneity of TME, we investigated the expression of BZW1 and BZW2 in stromal cell components. BZW1 was mainly expressed in B cells, fibroblasts and malignant cells, and BZW2 was upregulated in B cells, endothelial and malignant cells (Figures 8B,C).

### Expression of BZW1/2 in pancreatic cell lines

**FIGURE 9:**
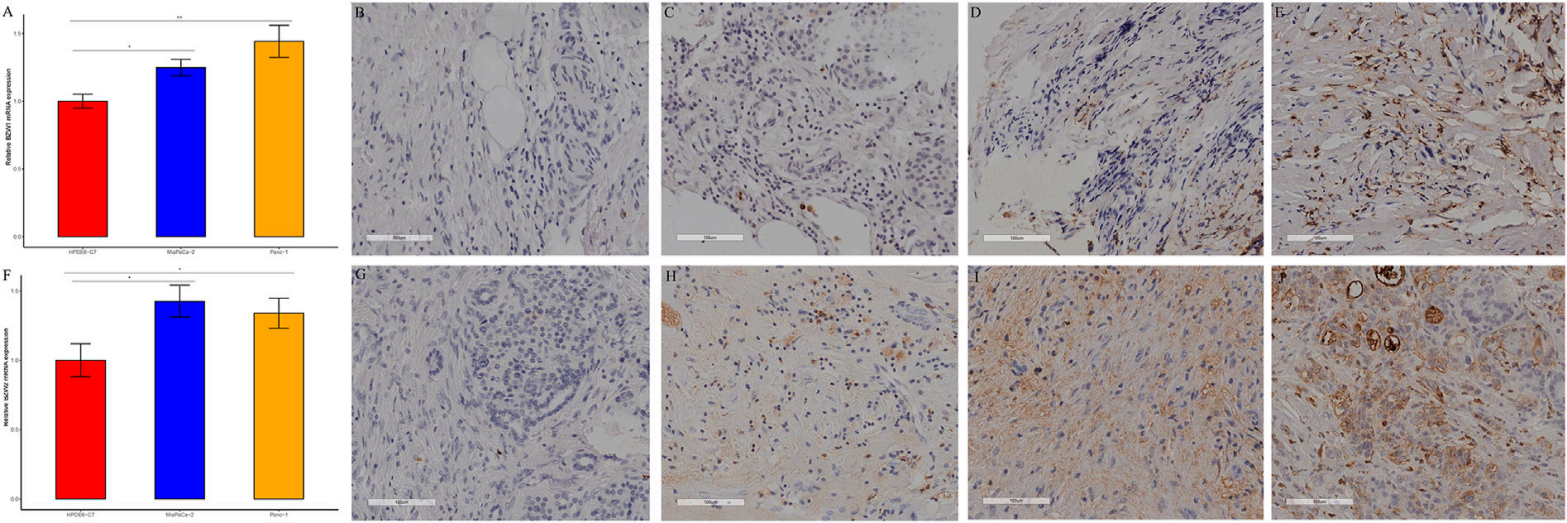
RT-qPCR and immunohistochemical staining verified the expression of BZW1 and BZW2 in PAAD samples. The relative mRNA expression levels of **(A)** BZW1 and **(F)** BZW2 in HPDE6-C7, MiaPaCa-2 and Panc-1 cell lines. **(B–E)** BZW1 was mainly expressed in the cell nucleus. Stain intensity: **(B)** BZW1, score 0. **(C)** BZW1, score 1. **(D)** BZW1, score 2. **(E)** BZW1, score 3. **(G–J)** BZW2 mainly expressed in cytoplasm and membrane. Stain intensity: **(G)** BZW2, score 0. **(H)** BZW2, score 1. **(I)** BZW2, score 2. **(J)** BZW2, score 3.

BZW1 and BZW2 expressions were further verified through RT-qPCR in HPDE6-C7, MiaPaCa-2 and Panc-1 cell lines. The mRNA levels of BZW1 and BZW2 were significantly higher in Panc-1 and MiaPaCa-2 cell lines than in the HPDE6-C7 cell line (Figures 9A,F).

###  External cohort validation confirmed that BZW1/2 expression could predict OS in PAAD

**FIGURE 10:**
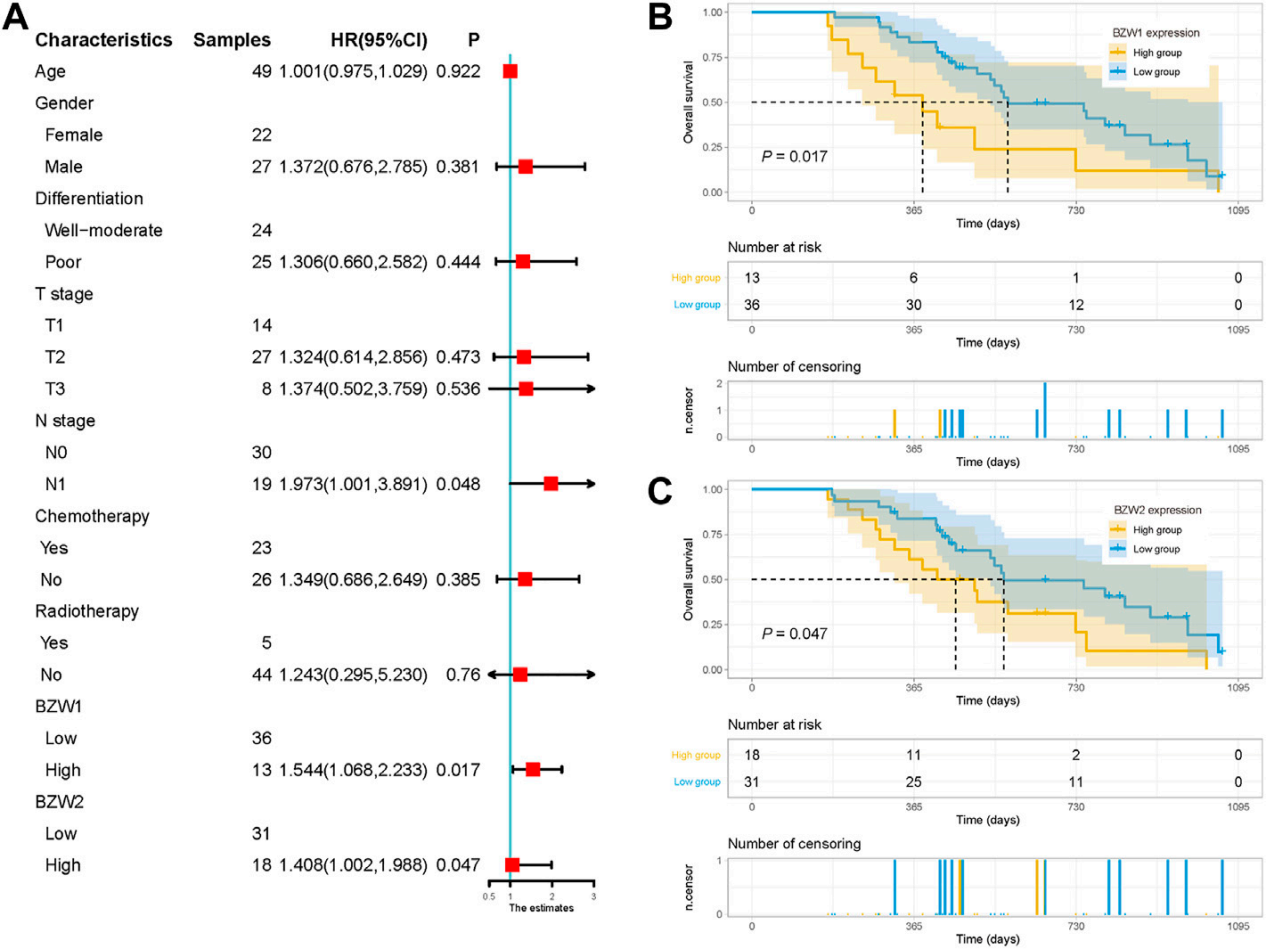
**(A)** Univariate analysis of OS in the external validation cohort. Survival curves for BZW1 **(B)** expression and BZW2 **(C)** expression in the external validation cohort.

49 samples were acquired as an external cohort, and all patients were successfully followed up. After immunohistochemical staining accomplished, the expression of BZW1 and BZW2 were classified as low- and high-expression based on scores of samples (Figure 9B-E, G-J). Among them, 13 and 36 patients were recognized as BZW1 high- and low-expression, respectively. 18 patients were identified as BZW2 high-expression, and 31 were low-expression. Correlations between BZW1/2 expression and clinicopathological factors of patients were listed in Supplementary Table S6. The expression of BZW1 and BZW2 showed no significant associations with age, gender, tumor location, differentiation, T stage, N stage, chemotherapy and radiotherapy. Survival analyses indicated that N stage, BZW1 expression and BZW2 expression were independent predictors for OS in PAAD patients (Figure 10A). High expression of BZW1 and BZW2 were significantly corrected with poor prognosis (BZW1, *p* = 0.017; BZW2, *p* = 0.047) (Figures 10B,C).

## Discussion and conclusion

In China, PAAD ranks the sixth leading cause of cancer related deaths (Xia et al., 2022). Notwithstanding that significant inroads had been achieved in recent years, PAAD patients still suffered poor prognoses and relatively short OS time (Xia et al., 2022). Given the insensitivity and drug-resistance, conventional therapy, which included surgery, chemotherapy and radiotherapy, often led to non-satisfactory curative effect nowadays. Targeted and immunological therapies were prized in the treatment of PAAD. Indeed, there was a long way to go for new therapies to be translated clinically. Based on publicly available tumor database, RNA-seq data could be used to search for prognostic-related genes and potential therapeutic targets. BZW1 and BZW2 were known as eukaryotic translation initiation factor 5 (eIF-5) mimic proteins (Singh et al., 2011; Loughran et al., 2018). It was reported that they shared a C-terminal W2 HEAT domain and an N-terminal bZIP domain (Aravind and Koonin, 2000; Loughran et al., 2018). In this study, we analyzed the expression levels of BZW1 and BZW2 in pan-cancer and distinct stages of PAAD based on the mRNA-seq data from TCGA databases. Our results suggested that BZW1 and BZW2 were highly expressed in PAAD tissue compared to normal tissue. Results of RT-qPCR indicated that the expression levels of BZW1 and BZW2 in human pancreatic cancer cell lines were higher than in the human pancreatic epithelium cell line. Immunohistochemical staining showed that BZW1 was mainly located in the nucleus, while BZW2 was mainly expressed in the cytoplasm and membrane. Survival analyses indicated that BZW2 expression was an independent predictor for OS of PAAD patients. A nomogram based on BZW2 expression and clinicopathological factors was established to predict the prognosis of PAAD.

Current evidence suggested that BZW1 and BZW2 could influence the stringency of start codon selection in mammalian cells (Loughran et al., 2018). Besides, BZW1 transcripts were alternatively polyadenylated and expressed in tissue-specific pattern (Yu et al., 2006). The functions of BZW1 and BZW2 had been largely understudied until recently. BZW1 and BZW2 have been established to promote the malignant progression of several cancers. Shi et al. indicated that BZW1 overexpression could promote prostate cancer cell proliferation by regulating TGF-β/Smad pathway (Shi et al., 2021). Jin et al. showed that overexpression of BZW2 in hepatocellular carcinoma cells significantly stimulated the activation of PI3K/AKT/mTOR pathway (Jin et al., 2019). Huang et al. demonstrated that BZW2 promoted malignant progression of colorectal cancer *via* activating ERK/MAPK pathway (Huang et al., 2020). Data presented in our study substantiated that high expression of BZW1 and BZW2 were predictors for poor prognosis of PAAD patients. Li et al. demonstrated that BZW1 could promote cell proliferation and inhibit apoptosis of PAAD cells *via* facilitating glycolysis in mouse xenograft models and organoids (Li et al., 2022). They inferred that BZW1 had the potential to be a new therapeutic target for PAAD (Li et al., 2022).

PPI network analysis in our study showed an interaction between BZW1 and BZW2, and single protein that connected both BZW1 and BZW2 included eIF1, eIF2S2, eIF5, SNX13 and AGR3. A broad range of eIFs had been established to regulate the initiation step of translation (Jackson et al., 2010). Members in the eIFs family were relevant to PAAD biology, and eIF1, eIF2D, eIF3C and eIF6 were identified as new biomarkers of PAAD (Golob-Schwarzl et al., 2020). Singh et al. indicated that BZW1 and BZW2 inhibited the recruitment and recycling of eIF2 by inhibiting its association with eIF5(Singh et al., 2011). Hiraishi et al. reported that BZW2 could serve as a competitor with eIF5, and BZW1 and BZW2 enhanced translation of ATF4, a key protein of endoplasmic reticulum stress and a potential target of PAAD (Hiraishi et al., 2014). Nachmias et al. proposed that BZW1 and BZW2 mediated the stemness and survival of leukemia stem cells (Nachmias et al., 2022). Moreover, BZW1 and BZW2 could regulate TYK2 expression, which acted as an oncogene and a therapeutic target in acute myelocytic leukemia (Sanda et al., 2013; Nachmias et al., 2022). Li et al. indicated that BZW1 facilitated eIF2α phosphorylation and promoted internal ribosome entry site-dependent translation of HIF1α and c-Myc in PAAD samples (Li et al., 2022).

To further clarify the roles of BZW1 and BZW2 in TME of PAAD, we conducted GSVA for each sample in TCGA database. The results revealed that BZW1 and BZW2 expression positively correlated with T cell mediated immune response to tumor cell. Correlations between BZW1/2 expression and TIICs were further analyzed. BZW1 and BZW2 expression were positively correlated with Th2 cells, which might facilitate tumor growth in TME of PAAD (Piro et al., 2017). Interestingly, TISCH analyses showed that BZW1 and BZW2 might regulate TME of PAAD by targeting B cells, which were activated by Th2 cells. Moreover, BZW1 positively correlated with Stromal score and ESTIMATE score. These results may reveal the potential roles of BZW1 and BZW2 in immune TME of PAAD.

Although a significant association with the prognosis of PAAD was found, BZW1 and BZW2 expression were not significantly correlated with gender, grade, AJCC stage, N stage and M stage. These results might indicate that the expression of BZW1/2 was relatively stable during tumor progression. However, several limitations were found in this study. First, subgroup analyses were not carried out due to the relatively small sample size. Therefore, the prediction performance of BZW1/2 remained unclear among different subgroups. Second, the associations between BZW1/2 expression and immune TME were not externally validated. Furthermore, future studies should investigate the role of BZW2 in tumor growth and progression of PAAD. As potential targets of PAAD, more experiments were needed to clarify the detailed mechanisms underlying the roles of BZW1/2 in PAAD.

In conclusion, we found that BZW1 and BZW2 were highly expressed in malignant cells and B cells in the TME of PAAD. BZW2 was an independent predictor for prognosis of PAAD. BZW1 and BZW2 expression were positively associated with T cell mediated immune response to tumor cell and Th2 cells in PAAD.

## Data Availability

The datasets presented in this study can be found in online repositories. The names of the repository/repositories and accession number(s) can be found in the article/Supplementary Material.
