# Supplementary material for: Expression, oncological and immunological characterizations of BZW1/2 in pancreatic adenocarcinoma
Source: Front Genet. 2022 Oct 4;13:1002673. doi: 10.3389/fgene.2022.1002673 (PMC9576853; doi:10.3389/fgene.2022.1002673)
Supplement: Supplementary file 1 [file Table1.DOCX]

Table S1 BZW1 and BZW2 expression levels in normal and tumor tissues among pan-cancer.

| Cancer type | BZW1 | | | BZW2 | | |
| --- | --- | --- | --- | --- | --- | --- |
|  | Normal | Tumor | *P* | Normal | Tumor | *P* |
| GBM | 4.37±1.31 | 6.89±0.56 | 2.1e-86 | 2.89±1.42 | 5.51±0.79 | 4.4e-84 |
| GBMLGG | 4.37±1.31 | 6.52±0.71 | 1.2e-245 | 2.89±1.42 | 4.98±0.77 | 3.1e-226 |
| LGG | 4.37±1.31 | 6.41±0.71 | 9.6e-202 | 2.89±1.42 | 4.82±0.69 | 1.3e-182 |
| BRCA | 6.42±0.67 | 7.22±0.98 | 2.7e-45 | 5.19±0.55 | 6.30±0.70 | 5.2e-96 |
| CESC | 5.57±0.65 | 6.31±0.98 | 1.4e-3 | 4.50±0.59 | 5.70±0.69 | 6.8e-7 |
| LUAD | 6.03±0.94 | 6.92±0.79 | 5.5e-66 | 4.61±0.93 | 5.95±0.68 | 4.5e-111 |
| ESCA | 5.95±1.50 | 6.84±0.67 | 2.5e-29 | 5.04±1.28 | 6.42±0.70 | 7.4e-72 |
| STES | 5.69±1.61 | 6.52±0.77 | 3.2e-45 | 4.97±1.36 | 6.17±0.76 | 4.8e-134 |
| COAD | 5.19±1.77 | 6.71±0.61 | 5.0e-77 | 4.89±1.66 | 6.62±0.58 | 9.5e-94 |
| COADREAD | 5.23±1.76 | 6.73±0.64 | 1.7e-83 | 4.92±1.64 | 6.63±0.57 | 8.8e-110 |
| PRAD | 6.00±0.84 | 6.34±0.93 | 3.6e-7 | 4.49±0.74 | 5.04±0.61 | 4.4e-17 |
| STAD | 4.87±1.69 | 6.38±0.77 | 4.6e-54 | 4.73±1.57 | 6.07±0.75 | 5.7e-56 |
| LUSC | 6.03±0.94 | 6.90±0.71 | 6.8e-71 | 4.61±0.93 | 6.25±0.62 | 4.7e-130 |
| LIHC | 5.28±0.80 | 5.58±0.98 | 4.5e-5 | 2.69±0.82 | 3.98±0.89 | 3.4e-41 |
| WT | 6.04±1.57 | 6.80±0.77 | 3.6e-10 | 4.30±1.34 | 6.00±0.60 | 3.3e-40 |
| SKCM | 5.16±0.50 | 5.73±1.26 | 1.4e-13 | 5.54±0.39 | 6.26±1.03 | 5.1e-19 |
| THCA | 5.60±1.01 | 5.70±0.82 | 2.7e-4 | 5.36±0.99 | 5.16±0.53 | 1.2e-8 |
| OV | 5.42±0.57 | 5.75±1.17 | 1.9e-6 | 4.11±0.45 | 5.44±1.07 | 3.0e-38 |
| PAAD | 3.60±1.59 | 6.28±0.91 | 6.6e-53 | 3.61±1.58 | 5.23±0.66 | 1.5e-46 |
| UCS | 5.42±0.58 | 6.13±0.82 | 5.0e-8 | 4.30±0.46 | 6.12±0.68 | 2.6e-21 |
| ALL | 3.71±1.28 | 6.26±0.93 | 2.9e-52 | 1.63±1.19 | 4.79±0.84 | 1.7e-60 |
| LAML | 3.71±1.28 | 6.69±0.56 | 1.6e-72 | 1.63±1.19 | 5.85±0.58 | 2.5e-76 |
| KIRP | 6.04±1.57 | 5.59±1.12 | 2.1e-8 | 4.30±1.34 | 4.65±0.73 | 6.1e-4 |
| KIPAN | 6.04±1.57 | 5.80±1.06 | 2.2e-5 | 4.30±1.34 | 4.32±0.76 | 0.03 |
| TGCT | 7.01±0.56 | 6.54±0.88 | 1.6e-7 | 3.78±0.43 | 5.30±0.89 | 5.6e-39 |
| PCPG | 6.72±0.90 | 5.30±0.95 | 0.03 | 4.37±0.72 | 4.05±0.76 | 0.37 |
| ACC | 5.88±1.50 | 5.58±1.21 | 6.9e-3 | 3.99±1.30 | 4.32±0.99 | 6.2e-3 |
| KICH | 4.92±1.13 | 6.04±1.57 | 1.5e-13 | 4.30±1.34 | 4.14±0.96 | 9.8e-4 |
| UCEC | 5.69±1.14 | 5.62±0.61 | 0.30 | 4.73±0.44 | 5.67±0.89 | 2.1e-7 |
| HNSC | 6.92±0.89 | 6.70±0.83 | 0.07 | 5.56±0.79 | 5.85±0.64 | 0.02 |
| KIRC | 6.02±0.93 | 6.04±1.57 | 0.06 | 4.30±1.34 | 4.17±0.69 | 1.0e-6 |
| BLCA | 5.98±0.97 | 6.22±0.63 | 0.31 | 5.86±0.42 | 6.14±0.71 | 0.01 |
| READ | 6.78±0.75 | 6.83±0.38 | 0.86 | 5.91±0.20 | 6.67±0.56 | 1.0e-5 |
| CHOL | 6.17±0.93 | 5.81±0.63 | 0.10 | 2.61±0.27 | 5.10±0.72 | 2.3e-9 |
