# Supplementary material for: Expression, oncological and immunological characterizations of BZW1/2 in pancreatic adenocarcinoma
Source: Front Genet. 2022 Oct 4;13:1002673. doi: 10.3389/fgene.2022.1002673 (PMC9576853; doi:10.3389/fgene.2022.1002673)
Supplement: Supplementary file 2 [file Table8.DOCX]

Table 1 Correlation of BZW1/2 expression and ESTIMATE-Stromal-Immune score (Pearson)

| Gene symbol | Stromal score | | Immune score | | ESTIMATE score | |
| --- | --- | --- | --- | --- | --- | --- |
|  | R value | *P* value | R value | *P* value | R value | *P* value |
| BZW1 | 0.253 | 6.57*E*-4 | 0.078 | 0.301 | 0.173 | 0.021 |
| BZW2 | -0.030 | 0.689 | -0.029 | 0.698 | -0.031 | 0.678 |
