# Supplementary material for: Expression, oncological and immunological characterizations of BZW1/2 in pancreatic adenocarcinoma
Source: Front Genet. 2022 Oct 4;13:1002673. doi: 10.3389/fgene.2022.1002673 (PMC9576853; doi:10.3389/fgene.2022.1002673)
Supplement: Supplementary file 5 [file Table7.DOCX]

Table S7 Acronyms referred in the article

| Abbreviations | Full names |
| --- | --- |
| PAAD | pancreatic adenocarcinoma |
| TME | tumor microenvironment |
| BZW1 | Basic leucine zipper and W2 domain-containing protein 1 |
| BZW2 | Basic leucine zipper and W2 domain-containing protein 2 |
| TCGA | the Cancer Genome Atlas |
| GEO | Gene Expression Omnibus |
| HPA | Human Protein Atlas |
| CPTAC | clinical proteomic tumor or analysis consortium |
| AJCC | American Joint Committee on cancer |
| OS | Overall survival |
| GO | Gene ontology |
| KEGG | Kyoto Encyclopedia of Genes and Genomes |
| DAVID | Database for Annotation, Visualization, and Integrated Discovery |
| CC | Cellular component |
| MF | Molecular function |
| BP | Biological process |
| GSVA | Gene set variation analysis |
| MsigDB | Molecular Signatures Database |
| PPI | Protein-protein interaction |
| TISCH | Tumor Immune Single-Cell Hub |
| ESTIMATE | Estimation of Stromal and Immune Cells in Malignant Tumors Tissues using Expression Data |
| TIIC | tumor infiltrating immune cell |
| GBM | Glioblastoma multiforme |
| GBMLGG | Glioma |
| LGG | Brain Lower Grade Glioma |
| BRCA | Breast invasive carcinoma |
| CESC | Cervical squamous cell carcinoma and endocervical adenocarcinoma |
| LUAD | Lung adenocarcinoma |
| ESCA | Esophageal carcinoma |
| STES | Stomach and Esophageal carcinoma |
| COAD | Colon adenocarcinoma |
| COADREAD | Colon adenocarcinoma/Rectum adenocarcinoma |
| PRAD | Prostate adenocarcinoma |
| STAD | Stomach adenocarcinoma |
| LUSC | Lung squamous cell carcinoma |
| LIHC | Liver hepatocellular carcinoma |
| WT | High-Risk Wilms Tumor |
| THCA | Thyroid carcinoma |
| OV | Ovarian serous cystadenocarcinoma |
| UCS | Uterine Carcinosarcoma |
| ALL | Acute Lymphoblastic Leukemia |
| LAML | Acute Myeloid Leukemia |
| KIRP | Kidney renal papillary cell carcinoma |
| KIPAN | Pan-kidney cohort (KICH+KIRC+KIRP) |
| TGCT | Testicular Germ Cell Tumors |
| PCPG | Pheochromocytoma and Paraganglioma |
| ACC | Adrenocortical carcinoma |
| KICH | Kidney Chromophobe |
| UCEC | Uterine Corpus Endometrial Carcinoma |
| HNSC | Head and Neck squamous cell carcinoma |
| SKCM | Skin Cutaneous Melanoma |
| BLCA | Bladder Urothelial Carcinoma |
| READ | Rectum adenocarcinoma |
| CHOL | Cholangiocarcinoma |
| eIF | eukaryotic translation initiation factor |
