# Supplementary material for: Expression, oncological and immunological characterizations of BZW1/2 in pancreatic adenocarcinoma
Source: Front Genet. 2022 Oct 4;13:1002673. doi: 10.3389/fgene.2022.1002673 (PMC9576853; doi:10.3389/fgene.2022.1002673)
Supplement: Supplementary file 7 [file Table9.DOCX]

Table 2 Correlations between BZW1/2 expression and TIICs in PAAD (Pearson)

|  | Negative correlation | | | Positive correlation | | |
| --- | --- | --- | --- | --- | --- | --- |
|  | Cell type (dataset) | *P* value | R value | Cell type (dataset) | *P* value | R value |
| BZW1 | Basophils (xCELL) | 3.394E-9 | -0.426 | CLP (xCELL) | 1.718E-6 | 0.351 |
|  | CD4+ Tcm (xCELL) | 5.390E-7 | -0.366 | smooth muscle (xCELL) | 3.227E-8 | 0.401 |
|  | MEP (xCELL) | 1.155E-13 | -0.520 | Th2 cells (xCELL) | 2.804E-6 | 0.344 |
|  | NKT (xCELL) | 1.937E-08 | -0.407 | neutrophils (MCPcounter) | 4.127E-8 | 0.398 |
|  | Th1 cells (xCELL) | 7.521E-13 | -0.505 | neutrophils (quanTIseq) | 4.962E-9 | 0.422 |
|  |  |  |  | neutrophils (TIMER) | 7.93E-10 | 0.441 |
|  |  |  |  | T cell CD8 (TIMER) | 1.450E-16 | 0.569 |
|  |  |  |  | DC (TIMER) | 1.655E-15 | 0.552 |
|  |  |  |  | macrophages M1 (quanTIseq) | 2.030E-8 | 0.406 |
| BZW2 | hepatocytes (xCELL) | 2.062E-10 | -0.455 | epithelial cells (xCELL) | 5.393E-15 | 0.544 |
|  | neurons (xCELL) | 2.880E-10 | -0.451 | keratinocytes (xCELL) | 3.884E-10 | 0.448 |
|  | Tgd cells (xCELL) | 9.534E-6 | -0.326 | sebocytes (xCELL) | 1.122E-8 | 0.413 |
|  |  |  |  | Th2 cells (xCELL) | 2.049E-7 | 0.379 |
