# Supplementary material for: Expression, oncological and immunological characterizations of BZW1/2 in pancreatic adenocarcinoma
Source: Front Genet. 2022 Oct 4;13:1002673. doi: 10.3389/fgene.2022.1002673 (PMC9576853; doi:10.3389/fgene.2022.1002673)
Supplement: Supplementary file 8 [file Table2.DOCX]

Table S2 BZW1 and BZW2 expression levels in normal and tumor tissues from GEO database

| Cancer type | BZW1 | | | BZW2 | | |
| --- | --- | --- | --- | --- | --- | --- |
|  | Normal | Tumor | *P* | Normal | Tumor | *P* |
| GSE28735 | 7.95±0.45 | 8.51±0.46 | 4.0e-8 | 5.06±0.43 | 5.36±0.32 | 8.4e-5 |
| GSE62452 | 7.70±0.53 | 8.26±0.52 | 7.0e-9 | 4.83±0.43 | 5.05±0.34 | 2.5e-4 |
