# Supplementary material for: Expression, oncological and immunological characterizations of BZW1/2 in pancreatic adenocarcinoma
Source: Front Genet. 2022 Oct 4;13:1002673. doi: 10.3389/fgene.2022.1002673 (PMC9576853; doi:10.3389/fgene.2022.1002673)
Supplement: Supplementary file 9 [file Table5.DOCX]

Table S5 The results of protein-protein interaction, co-expression and co-occurrence.

| #node1 | node2 | node1_string_id | node2_string_id | neighborhood_on_chromosome | gene_fusion | phylogenetic_cooccurrence | homology | coexpression | experimentally_determined_interaction | database_annotated | automated_textmining | combined_score |
| --- | --- | --- | --- | --- | --- | --- | --- | --- | --- | --- | --- | --- |
| BZW1 | EIF5 | 9606.ENSP00000394316 | 9606.ENSP00000216554 | 0 | 0 | 0 | 0 | 0.242 | 0.346 | 0 | 0.489 | 0.725 |
| BZW1 | EIF2S3 | 9606.ENSP00000394316 | 9606.ENSP00000253039 | 0 | 0 | 0 | 0 | 0.304 | 0 | 0 | 0.227 | 0.439 |
| BZW1 | EIF2S1 | 9606.ENSP00000394316 | 9606.ENSP00000256383 | 0 | 0 | 0 | 0 | 0.321 | 0 | 0 | 0.189 | 0.426 |
| BZW1 | UBP1 | 9606.ENSP00000394316 | 9606.ENSP00000283629 | 0 | 0 | 0 | 0 | 0 | 0 | 0 | 0.596 | 0.596 |
| BZW1 | C2orf47 | 9606.ENSP00000394316 | 9606.ENSP00000295079 | 0 | 0 | 0 | 0 | 0.063 | 0 | 0 | 0.421 | 0.434 |
| BZW1 | LZTFL1 | 9606.ENSP00000394316 | 9606.ENSP00000296135 | 0 | 0 | 0 | 0 | 0 | 0 | 0 | 0.622 | 0.622 |
| BZW1 | TMEM43 | 9606.ENSP00000394316 | 9606.ENSP00000303992 | 0 | 0 | 0 | 0 | 0 | 0.593 | 0 | 0 | 0.593 |
| BZW1 | AGR3 | 9606.ENSP00000394316 | 9606.ENSP00000308606 | 0 | 0 | 0 | 0 | 0 | 0 | 0 | 0.404 | 0.404 |
| BZW1 | C2orf69 | 9606.ENSP00000394316 | 9606.ENSP00000312770 | 0 | 0 | 0 | 0 | 0.062 | 0 | 0 | 0.467 | 0.479 |
| BZW1 | TYW5 | 9606.ENSP00000394316 | 9606.ENSP00000346627 | 0 | 0 | 0 | 0 | 0 | 0 | 0 | 0.76 | 0.76 |
| BZW1 | KCTD18 | 9606.ENSP00000394316 | 9606.ENSP00000352941 | 0 | 0 | 0 | 0 | 0 | 0 | 0 | 0.679 | 0.679 |
| BZW1 | ETF1 | 9606.ENSP00000394316 | 9606.ENSP00000353741 | 0 | 0 | 0 | 0 | 0.37 | 0.272 | 0 | 0.202 | 0.602 |
| BZW1 | EIF3I | 9606.ENSP00000394316 | 9606.ENSP00000362688 | 0 | 0 | 0 | 0 | 0.342 | 0 | 0 | 0.132 | 0.405 |
| BZW1 | AOX1 | 9606.ENSP00000394316 | 9606.ENSP00000363832 | 0 | 0 | 0 | 0 | 0 | 0 | 0 | 0.432 | 0.432 |
| BZW1 | EIF2S2 | 9606.ENSP00000394316 | 9606.ENSP00000364119 | 0 | 0 | 0 | 0 | 0.232 | 0.171 | 0 | 0.53 | 0.675 |
| BZW1 | SNX13 | 9606.ENSP00000394316 | 9606.ENSP00000398789 | 0 | 0 | 0 | 0 | 0 | 0 | 0 | 0.496 | 0.495 |
| BZW1 | EIF1 | 9606.ENSP00000394316 | 9606.ENSP00000419449 | 0 | 0 | 0 | 0 | 0.092 | 0 | 0 | 0.511 | 0.537 |
| BZW1 | SPATS2L | 9606.ENSP00000394316 | 9606.ENSP00000482515 | 0 | 0 | 0 | 0 | 0 | 0 | 0 | 0.748 | 0.748 |
| BZW1 | BZW2 | 9606.ENSP00000394316 | 9606.ENSP00000397249 | 0 | 0 | 0 | 0.976 | 0.095 | 0.793 | 0 | 0.694 | 0.808 |
| BZW2 | EIF5 | 9606.ENSP00000397249 | 9606.ENSP00000216554 | 0 | 0 | 0 | 0 | 0.098 | 0.346 | 0 | 0.567 | 0.722 |
| BZW2 | AHR | 9606.ENSP00000397249 | 9606.ENSP00000242057 | 0 | 0 | 0 | 0 | 0 | 0 | 0 | 0.598 | 0.598 |
| BZW2 | TSPAN13 | 9606.ENSP00000397249 | 9606.ENSP00000262067 | 0 | 0 | 0 | 0 | 0.076 | 0 | 0 | 0.788 | 0.796 |
| BZW2 | ANKMY2 | 9606.ENSP00000397249 | 9606.ENSP00000303570 | 0 | 0 | 0 | 0 | 0.108 | 0 | 0 | 0.835 | 0.847 |
| BZW2 | AGR3 | 9606.ENSP00000397249 | 9606.ENSP00000308606 | 0 | 0 | 0 | 0 | 0 | 0 | 0 | 0.759 | 0.759 |
| BZW2 | EIF2S2 | 9606.ENSP00000397249 | 9606.ENSP00000364119 | 0 | 0 | 0 | 0 | 0.112 | 0.534 | 0 | 0.549 | 0.797 |
| BZW2 | BZW1 | 9606.ENSP00000397249 | 9606.ENSP00000394316 | 0 | 0 | 0 | 0.976 | 0.095 | 0.793 | 0 | 0.694 | 0.808 |
| BZW2 | EIF2B1 | 9606.ENSP00000397249 | 9606.ENSP00000416250 | 0 | 0 | 0 | 0 | 0.061 | 0.466 | 0 | 0.062 | 0.488 |
| BZW2 | FKBP8 | 9606.ENSP00000397249 | 9606.ENSP00000476767 | 0 | 0 | 0 | 0 | 0.066 | 0 | 0 | 0.525 | 0.538 |
| BZW2 | EIF1 | 9606.ENSP00000397249 | 9606.ENSP00000419449 | 0 | 0 | 0 | 0 | 0.062 | 0 | 0 | 0.536 | 0.547 |
| BZW2 | SNX13 | 9606.ENSP00000397249 | 9606.ENSP00000398789 | 0 | 0 | 0 | 0 | 0 | 0 | 0 | 0.81 | 0.81 |
| AGR3 | BZW1 | 9606.ENSP00000308606 | 9606.ENSP00000394316 | 0 | 0 | 0 | 0 | 0 | 0 | 0 | 0.404 | 0.404 |
| AGR3 | BZW2 | 9606.ENSP00000308606 | 9606.ENSP00000397249 | 0 | 0 | 0 | 0 | 0 | 0 | 0 | 0.759 | 0.759 |
| AHR | BZW2 | 9606.ENSP00000242057 | 9606.ENSP00000397249 | 0 | 0 | 0 | 0 | 0 | 0 | 0 | 0.598 | 0.598 |
| ANKMY2 | BZW2 | 9606.ENSP00000303570 | 9606.ENSP00000397249 | 0 | 0 | 0 | 0 | 0.108 | 0 | 0 | 0.835 | 0.847 |
| AOX1 | BZW1 | 9606.ENSP00000363832 | 9606.ENSP00000394316 | 0 | 0 | 0 | 0 | 0 | 0 | 0 | 0.432 | 0.432 |
| BZW1 | BZW2 | 9606.ENSP00000394316 | 9606.ENSP00000397249 | 0 | 0 | 0 | 0.976 | 0.095 | 0.793 | 0 | 0.694 | 0.808 |
| BZW2 | BZW1 | 9606.ENSP00000397249 | 9606.ENSP00000394316 | 0 | 0 | 0 | 0.976 | 0.095 | 0.793 | 0 | 0.694 | 0.808 |
| C2orf47 | BZW1 | 9606.ENSP00000295079 | 9606.ENSP00000394316 | 0 | 0 | 0 | 0 | 0.063 | 0 | 0 | 0.421 | 0.434 |
| C2orf69 | BZW1 | 9606.ENSP00000312770 | 9606.ENSP00000394316 | 0 | 0 | 0 | 0 | 0.062 | 0 | 0 | 0.467 | 0.479 |
| EIF1 | BZW1 | 9606.ENSP00000419449 | 9606.ENSP00000394316 | 0 | 0 | 0 | 0 | 0.092 | 0 | 0 | 0.511 | 0.537 |
| EIF1 | BZW2 | 9606.ENSP00000419449 | 9606.ENSP00000397249 | 0 | 0 | 0 | 0 | 0.062 | 0 | 0 | 0.536 | 0.547 |
| EIF2B1 | BZW2 | 9606.ENSP00000416250 | 9606.ENSP00000397249 | 0 | 0 | 0 | 0 | 0.061 | 0.466 | 0 | 0.062 | 0.488 |
| EIF2S1 | BZW1 | 9606.ENSP00000256383 | 9606.ENSP00000394316 | 0 | 0 | 0 | 0 | 0.321 | 0 | 0 | 0.189 | 0.426 |
| EIF2S2 | BZW1 | 9606.ENSP00000364119 | 9606.ENSP00000394316 | 0 | 0 | 0 | 0 | 0.232 | 0.171 | 0 | 0.53 | 0.675 |
| EIF2S2 | BZW2 | 9606.ENSP00000364119 | 9606.ENSP00000397249 | 0 | 0 | 0 | 0 | 0.112 | 0.534 | 0 | 0.549 | 0.797 |
| EIF2S3 | BZW1 | 9606.ENSP00000253039 | 9606.ENSP00000394316 | 0 | 0 | 0 | 0 | 0.304 | 0 | 0 | 0.227 | 0.439 |
| EIF3I | BZW1 | 9606.ENSP00000362688 | 9606.ENSP00000394316 | 0 | 0 | 0 | 0 | 0.342 | 0 | 0 | 0.132 | 0.405 |
| EIF5 | BZW2 | 9606.ENSP00000216554 | 9606.ENSP00000397249 | 0 | 0 | 0 | 0 | 0.098 | 0.346 | 0 | 0.567 | 0.722 |
| EIF5 | BZW1 | 9606.ENSP00000216554 | 9606.ENSP00000394316 | 0 | 0 | 0 | 0 | 0.242 | 0.346 | 0 | 0.489 | 0.725 |
| ETF1 | BZW1 | 9606.ENSP00000353741 | 9606.ENSP00000394316 | 0 | 0 | 0 | 0 | 0.37 | 0.272 | 0 | 0.202 | 0.602 |
| FKBP8 | BZW2 | 9606.ENSP00000476767 | 9606.ENSP00000397249 | 0 | 0 | 0 | 0 | 0.066 | 0 | 0 | 0.525 | 0.538 |
| KCTD18 | BZW1 | 9606.ENSP00000352941 | 9606.ENSP00000394316 | 0 | 0 | 0 | 0 | 0 | 0 | 0 | 0.679 | 0.679 |
| LZTFL1 | BZW1 | 9606.ENSP00000296135 | 9606.ENSP00000394316 | 0 | 0 | 0 | 0 | 0 | 0 | 0 | 0.622 | 0.622 |
| SNX13 | BZW1 | 9606.ENSP00000398789 | 9606.ENSP00000394316 | 0 | 0 | 0 | 0 | 0 | 0 | 0 | 0.496 | 0.495 |
| SNX13 | BZW2 | 9606.ENSP00000398789 | 9606.ENSP00000397249 | 0 | 0 | 0 | 0 | 0 | 0 | 0 | 0.81 | 0.81 |
| SPATS2L | BZW1 | 9606.ENSP00000482515 | 9606.ENSP00000394316 | 0 | 0 | 0 | 0 | 0 | 0 | 0 | 0.748 | 0.748 |
| TMEM43 | BZW1 | 9606.ENSP00000303992 | 9606.ENSP00000394316 | 0 | 0 | 0 | 0 | 0 | 0.593 | 0 | 0 | 0.593 |
| TSPAN13 | BZW2 | 9606.ENSP00000262067 | 9606.ENSP00000397249 | 0 | 0 | 0 | 0 | 0.076 | 0 | 0 | 0.788 | 0.796 |
| TYW5 | BZW1 | 9606.ENSP00000346627 | 9606.ENSP00000394316 | 0 | 0 | 0 | 0 | 0 | 0 | 0 | 0.76 | 0.76 |
| UBP1 | BZW1 | 9606.ENSP00000283629 | 9606.ENSP00000394316 | 0 | 0 | 0 | 0 | 0 | 0 | 0 | 0.596 | 0.596 |
