# Supplementary material for: Expression, oncological and immunological characterizations of BZW1/2 in pancreatic adenocarcinoma
Source: Front Genet. 2022 Oct 4;13:1002673. doi: 10.3389/fgene.2022.1002673 (PMC9576853; doi:10.3389/fgene.2022.1002673)
Supplement: Supplementary file 10 [file Table3.DOCX]

Table S3 Relationships between BZW1/2 expression and clinicopathological parameters of PAAD

|  |  | BZW1 | *P* | BZW2 | *P* |
| --- | --- | --- | --- | --- | --- |
| Gender | Female(N=81) | 6.25±0.96 | 0.50 | 5.23±0.76 | 0.83 |
|  | Male(N=83) | 6.34±0.79 |  | 5.25±0.58 |  |
| Grade | G1(N=4) | 6.61±0.31 | 0.67 | 5.28±0.76 | 0.99 |
|  | G2(N=18) | 6.46±0.73 |  | 5.29±0.63 |  |
|  | G3(N=34) | 6.31±0.82 |  | 5.31±0.62 |  |
| Stage | I(N=47) | 6.33±1.11 | 0.88 | 5.24±0.78 | 0.84 |
|  | II(N=25) | 6.32±0.91 |  | 5.37±0.50 |  |
|  | III(N=32) | 6.21±0.70 |  | 5.21±0.72 |  |
|  | IV(N=20) | 6.42±0.63 |  | 5.25±0.60 |  |
| T | T1(N=37) | 6.38±1.00 | 0.39 | 5.27±0.78 | <0.05 |
|  | T2(N=35) | 6.34±0.75 |  | 5.33±0.48 |  |
|  | T3(N=37) | 6.11±0.82 |  | 4.98±0.76 |  |
|  | T4(N=15) | 6.49±0.47 |  | 5.49±0.58 |  |
| N | N0(N=69) | 6.37±0.90 | 0.87 | 5.29±0.70 | 0.61 |
|  | N1(N=30) | 6.30±0.74 |  | 5.17±0.71 |  |
|  | N2(N=9) | 6.45±0.50 |  | 5.41±0.41 |  |
| M | M0(N=100) | 6.34±0.85 | 0.69 | 5.25±0.70 | 0.68 |
|  | M1(N=9) | 6.24±0.67 |  | 5.15±0.67 |  |
