# Supplementary material for: Expression, oncological and immunological characterizations of BZW1/2 in pancreatic adenocarcinoma
Source: Front Genet. 2022 Oct 4;13:1002673. doi: 10.3389/fgene.2022.1002673 (PMC9576853; doi:10.3389/fgene.2022.1002673)
Supplement: Supplementary file 11 [file Table4.DOCX]

Table S4. The PCC values of the top 100 similar genes of BZW1 and BZW2 in GEPIA webtool.

|  | Gene Symbol | Gene ID | PCC |
| --- | --- | --- | --- |
| BZW1 | BZW1P2 | ENSG00000198406.7 | 0.89 |
|  | NAA50 | ENSG00000121579.12 | 0.81 |
|  | KPNA4 | ENSG00000186432.8 | 0.8 |
|  | G2E3 | ENSG00000092140.14 | 0.8 |
|  | RAB10 | ENSG00000084733.10 | 0.78 |
|  | NMD3 | ENSG00000169251.12 | 0.78 |
|  | WDFY1 | ENSG00000085449.14 | 0.77 |
|  | PTBP3 | ENSG00000119314.15 | 0.77 |
|  | PPP2R5E | ENSG00000154001.13 | 0.77 |
|  | MAPK8 | ENSG00000107643.15 | 0.77 |
|  | KPNA1 | ENSG00000114030.12 | 0.77 |
|  | ITCH | ENSG00000078747.12 | 0.77 |
|  | COMMD2 | ENSG00000114744.8 | 0.77 |
|  | YY1 | ENSG00000100811.10 | 0.76 |
|  | TMEM39A | ENSG00000176142.12 | 0.76 |
|  | RPE | ENSG00000197713.14 | 0.76 |
|  | OPA1 | ENSG00000198836.8 | 0.76 |
|  | MTDH | ENSG00000147649.9 | 0.76 |
|  | TOX4 | ENSG00000092203.13 | 0.75 |
|  | SPTY2D1 | ENSG00000179119.14 | 0.75 |
|  | PRPF40A | ENSG00000196504.15 | 0.75 |
|  | NAA15 | ENSG00000164134.12 | 0.75 |
|  | MTPN | ENSG00000105887.10 | 0.75 |
|  | KCMF1 | ENSG00000176407.17 | 0.75 |
|  | EIF2S1 | ENSG00000134001.12 | 0.75 |
|  | CAB39 | ENSG00000135932.10 | 0.75 |
|  | YWHAG | ENSG00000170027.6 | 0.74 |
|  | UBE2D3 | ENSG00000109332.19 | 0.74 |
|  | SEC24A | ENSG00000113615.12 | 0.74 |
|  | RBM7 | ENSG00000076053.10 | 0.74 |
|  | MARCH7 | ENSG00000136536.14 | 0.74 |
|  | MAPK6 | ENSG00000069956.11 | 0.74 |
|  | FCF1 | ENSG00000119616.11 | 0.74 |
|  | EIF4E | ENSG00000151247.12 | 0.74 |
|  | DDX18 | ENSG00000088205.12 | 0.74 |
|  | CAPZA2 | ENSG00000198898.12 | 0.74 |
|  | ATP13A3 | ENSG00000133657.14 | 0.74 |
|  | ATP11B | ENSG00000058063.15 | 0.74 |
|  | ARIH1 | ENSG00000166233.12 | 0.74 |
|  | ACTR3 | ENSG00000115091.11 | 0.74 |
|  | ABCE1 | ENSG00000164163.10 | 0.74 |
|  | ZFP91 | ENSG00000186660.14 | 0.73 |
|  | YWHAZP4 | ENSG00000213131.3 | 0.73 |
|  | YME1L1 | ENSG00000136758.18 | 0.73 |
|  | XPOT | ENSG00000184575.11 | 0.73 |
|  | USP37 | ENSG00000135913.10 | 0.73 |
|  | UBA6 | ENSG00000033178.12 | 0.73 |
|  | TMOD3 | ENSG00000138594.12 | 0.73 |
|  | SENP2 | ENSG00000163904.12 | 0.73 |
|  | PPP2CA | ENSG00000113575.9 | 0.73 |
|  | PGM2 | ENSG00000169299.13 | 0.73 |
|  | NDUFS1 | ENSG00000023228.13 | 0.73 |
|  | IPO7 | ENSG00000205339.9 | 0.73 |
|  | GORASP2 | ENSG00000115806.12 | 0.73 |
|  | FNDC3B | ENSG00000075420.12 | 0.73 |
|  | EXT1 | ENSG00000182197.10 | 0.73 |
|  | ESYT2 | ENSG00000117868.15 | 0.73 |
|  | DR1 | ENSG00000117505.12 | 0.73 |
|  | DEK | ENSG00000124795.14 | 0.73 |
|  | COPB2 | ENSG00000184432.9 | 0.73 |
|  | ARHGAP5 | ENSG00000100852.12 | 0.73 |
|  | AGFG1 | ENSG00000173744.17 | 0.73 |
|  | ADAM10 | ENSG00000137845.14 | 0.73 |
|  | XRCC5 | ENSG00000079246.15 | 0.72 |
|  | UBQLN1 | ENSG00000135018.13 | 0.72 |
|  | TROVE2 | ENSG00000116747.12 | 0.72 |
|  | TRA2B | ENSG00000136527.17 | 0.72 |
|  | TOP1 | ENSG00000198900.5 | 0.72 |
|  | SNX6 | ENSG00000129515.18 | 0.72 |
|  | SLAIN2 | ENSG00000109171.14 | 0.72 |
|  | SELT | ENSG00000198843.12 | 0.72 |
|  | RNASEH1 | ENSG00000171865.9 | 0.72 |
|  | RALB | ENSG00000144118.13 | 0.72 |
|  | RAB6A | ENSG00000175582.19 | 0.72 |
|  | PPP4R2 | ENSG00000163605.14 | 0.72 |
|  | NUP160 | ENSG00000030066.13 | 0.72 |
|  | NRAS | ENSG00000213281.4 | 0.72 |
|  | KLF7 | ENSG00000118263.14 | 0.72 |
|  | KIAA1715 | ENSG00000144320.13 | 0.72 |
|  | IMPAD1 | ENSG00000104331.8 | 0.72 |
|  | HNRNPK | ENSG00000165119.18 | 0.72 |
|  | GSPT1 | ENSG00000103342.12 | 0.72 |
|  | DCUN1D1 | ENSG00000043093.13 | 0.72 |
|  | CREB1 | ENSG00000118260.14 | 0.72 |
|  | CPSF2 | ENSG00000165934.12 | 0.72 |
|  | CNIH1 | ENSG00000100528.11 | 0.72 |
|  | CKAP5 | ENSG00000175216.14 | 0.72 |
|  | ASXL2 | ENSG00000143970.16 | 0.72 |
|  | AP1S3 | ENSG00000152056.16 | 0.72 |
|  | ANO6 | ENSG00000177119.15 | 0.72 |
|  | AGPS | ENSG00000018510.12 | 0.72 |
|  | ZFAND3 | ENSG00000156639.11 | 0.71 |
|  | TRIP12 | ENSG00000153827.13 | 0.71 |
|  | RBM12 | ENSG00000244462.7 | 0.71 |
|  | RAD23B | ENSG00000119318.12 | 0.71 |
|  | NCKAP1 | ENSG00000061676.14 | 0.71 |
|  | LSM12P1 | ENSG00000232024.2 | 0.71 |
|  | HMGXB4 | ENSG00000100281.13 | 0.71 |
|  | AP1G1 | ENSG00000166747.12 | 0.71 |
|  | ADAM17 | ENSG00000151694.12 | 0.71 |
| BZW2 | CBX3 | ENSG00000122565.18 | 0.72 |
|  | NIFK | ENSG00000155438.11 | 0.7 |
|  | AVL9 | ENSG00000105778.17 | 0.68 |
|  | PPIAP22 | ENSG00000198618.5 | 0.66 |
|  | PPIA | ENSG00000196262.13 | 0.66 |
|  | TBRG4 | ENSG00000136270.13 | 0.65 |
|  | CIRH1A | ENSG00000141076.17 | 0.65 |
|  | AIMP2 | ENSG00000106305.9 | 0.65 |
|  | HDGF | ENSG00000143321.18 | 0.65 |
|  | ZDHHC9 | ENSG00000188706.12 | 0.65 |
|  | GGCT | ENSG00000006625.17 | 0.64 |
|  | MRPL3 | ENSG00000114686.8 | 0.64 |
|  | EIF3B | ENSG00000106263.17 | 0.64 |
|  | LSM5 | ENSG00000106355.9 | 0.64 |
|  | WDR75 | ENSG00000115368.9 | 0.64 |
|  | FARSB | ENSG00000116120.9 | 0.64 |
|  | PLK1 | ENSG00000166851.14 | 0.63 |
|  | NUTF2 | ENSG00000102898.11 | 0.63 |
|  | NUDT5 | ENSG00000165609.12 | 0.63 |
|  | CCDC58 | ENSG00000160124.9 | 0.63 |
|  | CCT6A | ENSG00000146731.10 | 0.63 |
|  | NUP37 | ENSG00000075188.8 | 0.63 |
|  | EIF2S2 | ENSG00000125977.6 | 0.63 |
|  | UTP14A | ENSG00000156697.12 | 0.62 |
|  | ARMC10 | ENSG00000170632.13 | 0.62 |
|  | EIF2AK1 | ENSG00000086232.12 | 0.62 |
|  | MRPL30 | ENSG00000185414.19 | 0.62 |
|  | COA4 | ENSG00000181924.6 | 0.62 |
|  | NOL10 | ENSG00000115761.15 | 0.62 |
|  | KARS | ENSG00000065427.14 | 0.62 |
|  | OSBPL3 | ENSG00000070882.12 | 0.62 |
|  | APH1A | ENSG00000117362.12 | 0.61 |
|  | EMC8 | ENSG00000131148.8 | 0.61 |
|  | NOB1 | ENSG00000141101.12 | 0.61 |
|  | PSMA2 | ENSG00000106588.10 | 0.61 |
|  | CYB5B | ENSG00000103018.16 | 0.61 |
|  | VDAC1 | ENSG00000213585.10 | 0.61 |
|  | GSPT1 | ENSG00000103342.12 | 0.61 |
|  | CHCHD3 | ENSG00000106554.11 | 0.61 |
|  | EIF6 | ENSG00000242372.6 | 0.61 |
|  | PPM1G | ENSG00000115241.10 | 0.61 |
|  | TOMM34 | ENSG00000025772.7 | 0.61 |
|  | EIF2A | ENSG00000144895.11 | 0.61 |
|  | WDR12 | ENSG00000138442.9 | 0.61 |
|  | ECT2 | ENSG00000114346.13 | 0.61 |
|  | EIF3M | ENSG00000149100.12 | 0.6 |
|  | DARS2 | ENSG00000117593.9 | 0.6 |
|  | NUDT21 | ENSG00000167005.13 | 0.6 |
|  | RAC1 | ENSG00000136238.17 | 0.6 |
|  | GTPBP4 | ENSG00000107937.18 | 0.6 |
|  | CYCS | ENSG00000172115.8 | 0.6 |
|  | UCK2 | ENSG00000143179.12 | 0.6 |
|  | MTIF2 | ENSG00000085760.14 | 0.6 |
|  | OLA1 | ENSG00000138430.15 | 0.6 |
|  | NCL | ENSG00000115053.15 | 0.6 |
|  | CLNS1A | ENSG00000074201.8 | 0.6 |
|  | PTMA | ENSG00000187514.14 | 0.6 |
|  | PHB | ENSG00000167085.11 | 0.59 |
|  | CCT5 | ENSG00000150753.11 | 0.59 |
|  | PRIM2 | ENSG00000146143.17 | 0.59 |
|  | DDX27 | ENSG00000124228.14 | 0.59 |
|  | SF3B3 | ENSG00000189091.12 | 0.59 |
|  | PDCD10 | ENSG00000114209.14 | 0.59 |
|  | ACTL6A | ENSG00000136518.16 | 0.59 |
|  | PSMD7 | ENSG00000103035.10 | 0.59 |
|  | UBE2I | ENSG00000103275.18 | 0.59 |
|  | HMBS | ENSG00000256269.6 | 0.59 |
|  | CENPN | ENSG00000166451.13 | 0.59 |
|  | CKS1B | ENSG00000173207.12 | 0.59 |
|  | SF3B6 | ENSG00000115128.6 | 0.58 |
|  | PA2G4 | ENSG00000170515.13 | 0.58 |
|  | ZNF239 | ENSG00000196793.13 | 0.58 |
|  | DNAJC2 | ENSG00000105821.14 | 0.58 |
|  | DAP3 | ENSG00000132676.15 | 0.58 |
|  | C2orf47 | ENSG00000162972.10 | 0.58 |
|  | BRIX1 | ENSG00000113460.12 | 0.58 |
|  | DHODH | ENSG00000102967.11 | 0.58 |
|  | HEATR1 | ENSG00000119285.10 | 0.58 |
|  | TIMM8A | ENSG00000126953.5 | 0.58 |
|  | SNRPD1 | ENSG00000167088.10 | 0.58 |
|  | LRPPRC | ENSG00000138095.18 | 0.58 |
|  | MTHFD1 | ENSG00000100714.15 | 0.58 |
|  | BUB1 | ENSG00000169679.14 | 0.58 |
|  | HNRNPF | ENSG00000169813.16 | 0.58 |
|  | CDK7 | ENSG00000134058.10 | 0.58 |
|  | MTCH2 | ENSG00000109919.9 | 0.58 |
|  | TNPO3 | ENSG00000064419.13 | 0.58 |
|  | FAM136A | ENSG00000035141.7 | 0.58 |
|  | UQCRC2 | ENSG00000140740.10 | 0.58 |
|  | ALG3 | ENSG00000214160.9 | 0.57 |
|  | GMPS | ENSG00000163655.15 | 0.57 |
|  | CDC123 | ENSG00000151465.13 | 0.57 |
|  | HSPE1 | ENSG00000115541.10 | 0.57 |
|  | GSS | ENSG00000100983.9 | 0.57 |
|  | HNRNPA3 | ENSG00000170144.18 | 0.57 |
|  | TPX2 | ENSG00000088325.15 | 0.57 |
|  | GTF2F2 | ENSG00000188342.11 | 0.57 |
|  | TOMM20 | ENSG00000173726.10 | 0.57 |
|  | PAICS | ENSG00000128050.8 | 0.57 |
|  | RRP9 | ENSG00000114767.6 | 0.57 |
