# Supplementary material for: Expression, oncological and immunological characterizations of BZW1/2 in pancreatic adenocarcinoma
Source: Front Genet. 2022 Oct 4;13:1002673. doi: 10.3389/fgene.2022.1002673 (PMC9576853; doi:10.3389/fgene.2022.1002673)
Supplement: Supplementary file 12 [file Table6.DOCX]

Table S6 Correlations between BZW1/2 expression and clinicopathological factors of PAAD patients

|  | BZW1 expression | | | BZW2 expression | | |
| --- | --- | --- | --- | --- | --- | --- |
|  | Low (N=36) | High (N=13) | *P* | Low (N=31) | High (N=18) | *P* |
| Age | 63.47±12.07 | 65.77±10.08 | 0.543 | 63.42±12.49 | 65.22±9.86 | 0.603 |
| Gender |  |  | 0.915 |  |  | 0.961 |
| Female | 16(44.4%) | 6(46.2%) |  | 14(45.2%) | 8(44.4%) |  |
| Male | 20(55.6%) | 7(53.8%) |  | 17(54.8%) | 10(55.6%) |  |
| Tumor location |  |  | 0.226 |  |  | 0.579 |
| Head | 26(72.2%) | 7(53.8%) |  | 20(64.5%) | 13(72.2%) |  |
| Body or tail | 10(27.8%) | 6(46.2%) |  | 11(35.5%) | 5(27.8%) |  |
| Differentiation |  |  | 0.682 |  |  | 0.913 |
| Well-moderate | 19(52.8%) | 6(46.2%) |  | 16(51.6%) | 9(50.0%) |  |
| Poor | 17(47.2%) | 7(53.8%) |  | 15(48.4%) | 9(50.0%) |  |
| T stage |  |  | 0.848 |  |  | 0.717 |
| T1 | 11(30.6%) | 3(23.1%) |  | 9(21.0%) | 5(27.8%) |  |
| T2 | 19(52.8%) | 8(61.5%) |  | 16(51.6%) | 11(61.1%) |  |
| T3 | 6(16.7%) | 2(15.4%) |  | 6(19.4%) | 2(11.1%) |  |
| N stage |  |  | 0.175 |  |  | 0.219 |
| N0 | 20(55.6%) | 10(76.9%) |  | 21(67.7%) | 9(50.0%) |  |
| N1 | 16(44.4%) | 3(23.1%) |  | 10(32.3%) | 9(50.0%) |  |
| Chemotherapy |  |  | 0.173 |  |  | 0.790 |
| Yes | 19(52.8%) | 4(30.8%) |  | 15(48.4%) | 8(44.4%) |  |
| No | 17(47.2%) | 9(68.2%) |  | 16(51.6%) | 10(55.6%) |  |
| Radiotherapy |  |  | 0.074 |  |  | 0.873 |
| Yes | 2(5.6%) | 3(23.1%) |  | 3(9.7%) | 2(11.1%) |  |
| No | 34(94.4%) | 10(76.9%) |  | 28(90.3%) | 16(88.9%) |  |
